# A Learning‐Rate Modulable and Reliable TiO*_x_* Memristor Array for Robust, Fast, and Accurate Neuromorphic Computing

**DOI:** 10.1002/advs.202201117

**Published:** 2022-06-05

**Authors:** Jingon Jang, Sanggyun Gi, Injune Yeo, Sanghyeon Choi, Seonghoon Jang, Seonggil Ham, Byunggeun Lee, Gunuk Wang

**Affiliations:** ^1^ KU‐KIST Graduate School of Converging Science and Technology Korea University 145, Anam‐ro, Seongbuk‐gu Seoul 02841 Republic of Korea; ^2^ School of Electrical Engineering and Computer Science Gwangju Institute of Science and Technology 123, Cheomdangwagi‐ro, Buk‐gu, Gwangju, Republic of Korea Buk‐gu 61005 Republic of Korea; ^3^ Department of Integrative Energy Engineering Korea University 145, Anam‐ro, Seongbuk‐gu Seoul 02841 Republic of Korea; ^4^ Center for Neuromorphic Engineering Korea Institute of Science and Technology 5, Hwarang‐ro 14‐gil, Seongbuk‐gu Seoul 02792 Republic of Korea

**Keywords:** artificial synapses, hardware implementation, memristors, neuromorphic computing, uniformity

## Abstract

Realization of memristor‐based neuromorphic hardware system is important to achieve energy efficient bigdata processing and artificial intelligence in integrated device system‐level. In this sense, uniform and reliable titanium oxide (TiO*_x_*) memristor array devices are fabricated to be utilized as constituent device element in hardware neural network, representing passive matrix array structure enabling vector‐matrix multiplication process between multisignal and trained synaptic weight. In particular, in situ convolutional neural network hardware system is designed and implemented using a multiple 25 × 25 TiO*_x_* memristor arrays and the memristor device parameters are developed to bring global constant voltage programming scheme for entire cells in crossbar array without any voltage tuning peripheral circuit such as transistor. Moreover, the learning rate modulation during in situ hardware training process is successfully achieved due to superior TiO*_x_* memristor performance such as threshold uniformity (≈2.7%), device yield (> 99%), repetitive stability (≈3000 spikes), low asymmetry value of ≈1.43, ambient stability (6 months), and nonlinear pulse response. The learning rate modulable fast‐converging in situ training based on direct memristor operation shows five times less training iterations and reduces training energy compared to the conventional hardware in situ training at ≈95.2% of classification accuracy.

## Graphical abstract

The uniform and reliable 25 × 25 TiOx memristor array device shows superior performance as threshold uniformity (≈2.7 %), device yield (> 99%), repetitive stability (≈3000 spikes), low asymmetry (≈1.43), ambient stability (6 months) and nonlinear pulse response. The integrated neuromorphic hardware system is implemented with 5 times less training cost than conventional system at ≈95.2% of accuracy.

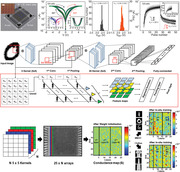

## 1 Introduction

The interest in neuromorphic computing hardware is grounded in the fact that it could overcome von Neumann bottleneck issues such as the memory wall and immense energy consumption generated during the movement of the enormous amount of data, which are major shortcomings in conventional computing architecture.^[^ ^1^, ^2^, ^3^, ^4^, ^5^, ^6^, ^7^, ^8^, ^9^, ^10^ ^]^ In particular, a memristor array capable of simple two‐terminal structure, nanoscale, and nonvolatile analog switching states can realize the ultimate area/energy savings in massively parallel computation, such as vector‐matrix multiplication (VMM), which is essential for neuromorphic computing. Recent trends in neuromorphic hardware implementation have highlighted these advantages of the memristor for fast and energy‐saving learning and recognition processes with an efficient training strategy.^[^ ^11^, ^12^, ^13^, ^14^, ^15^, ^16^, ^17^ ^]^ For example, Li et al.^[^ ^18^ ^]^ reported a large‐scale HfO_2_ memristor crossbar array for analog signal and image processing, Bayat et al.^[^ ^19^ ^]^ presented a hardware implementation of a bilayer memristor device for a multilayer perceptron network to recognize letter patterns, and Cai et al.^[^ ^20^ ^]^ actualized memristor‐integrated complementary metal‐oxide‐semiconductor circuits for a reprogrammable computing system.

However, major drawbacks of memristor devices such as switching degradation by repetitive programming pulse spikes, nonuniform switching features at the array level, and switching failure by low device yield are known to severely hinder optimal and efficient neuromorphic hardware implementation.^[^ ^21^, ^22^, ^23^ ^]^ For example, a lack of switching reliability and uniformity can result in each synaptic node being nonlinearly degraded or even stuck when the repetitive weight updating process proceeds. Furthermore, this nonuniform switching feature makes it difficult to properly update the synaptic weights to the desired values at the array level. These factors can cause severe performance and accuracy loss in neuromorphic hardware computing operation. To mitigate these issues, as the early stage for neuromorphic hardware implementation, an ex situ training method, in which a weight map trained from the software‐worked external computing unit is pre‐encoded to the conductance of the memristors, has been developed.^[^ ^24^, ^25^, ^26^ ^]^ However, this strategy is restricted by the fact that additional energy‐consuming computer resources for the training itself are indispensable and that the classification accuracy can be easily degraded because of the difficulty for the device switching imperfections to be reflected, such as endurance and retention. For these reasons, along with the recent improvement in the memristor, a hardware implementation of in situ training has been extensively reported based on their immunity for the device imperfections. This approach is based on a real‐time conductance adjustment at the system level through the error calculation between the target value and the VMM result.^[^ ^27^, ^28^, ^29^ ^]^ The capability of in situ training that updates the weight and performs VMM computation at the same hardware platform does not require extracting or encoding the weight between software and hardware, which enhances the area/energy efficiency ultimately.

To realize fast and precise in situ training with extreme area/energy savings, there are two requirements of the memristor in addition to resolving the aforementioned issues. First, the hardware complexity of peripheral circuits for conductance programming should be reduced. For example, in previous works, an on‐chip series transistor scheme (1T‐1R)^[^ ^28^, ^29^ ^]^ or precise tuning technique^[^ ^19^ ^]^ has been used to mitigate the arbitrary conductance change of nontarget devices caused by switching threshold variations of the memristor. Both methods require additional peripheral circuitry and processing to adjust the amplitude of the write pulse for every writing cycle, leading to a more complex and energy‐hungry process. Even equipment such as a parameter analyzer and a probe card used to be employed for reliable conductance writing, which makes it more difficult for a hardware system to be integrated on a board or chip as a fully functional system. Therefore, improving the switching threshold uniformity of memristors is essential for compact and energy‐efficient neuromorphic hardware implementation. Second, a massive number of iterations are required during the initial training phase in conventional in situ training, leading to a large increase in the training cost and time. To reduce the number of iterations, hybrid training has been suggested in previous works, in which the conductance is optimized via in situ training after transfer learning.^[^ ^30^, ^31^ ^]^ However, this approach could increase the entire training cost with the use of an external computing unit. Meanwhile, with regard to the software, optimization methods to the learning rate according to the progress of iteration, such as RMSprop,^[^ ^32^ ^]^ have been used mainly to reduce the required number of initial iterations.^[^ ^32^, ^33^, ^34^ ^]^ Inspired by this strategy, if the memristor is capable of changing the rate of conductance increase by optimizing the quantity of presynaptic pulses, it is expected that the learning rate can be adjusted according to the in situ training stages. There are several researches that use a method to find the optimal learning rate by adjusting the pulse conditions prior to training.^[^ ^20^, ^35^ ^]^ However, in order to implement the optimization method, the learning rate should be able to change during training, which requires a device with high switching uniformity. Note that improved switching characteristics and uniformity of the device ensure not only the stable writing in the passive array without tuning the pulse conditions for individual devices to compensate the device imperfections but also the optimal strategy to be applied by adjusting the learning rate during training for all devices in the array. In this regard, in addition to resolving their major drawbacks, as mentioned before, designing a memristor array that can satisfy switching threshold uniformity and the adjustment ability of the conductance change rate is necessary for optimal and advanced in situ training.^[^ ^36^, ^37^, ^38^, ^39^ ^]^

In this article, we show that the convolutional kernels using passive memristor arrays with high uniformity are in situ trained in hardware platform and the fast‐converging training is achieved by adjusting the conductance change rate of the memristor at the first time. Here, a 25 × 25 memristor crossbar array is developed using a stoichiometrically stabilized binary metal‐oxide memristive material, that is, titanium oxide (TiO*_x_*). The array presents >99% working device yield, an ≈2.7% switching threshold variation, extremely low asymmetry value of ≈1.43, and repetitive synaptic functional stability for every ≈3000 presynaptic pulse trains during ≈6 months of elapsed time. In particular, the designed TiO*_x_* memristor can clearly exhibit a nonlinear response according to the amplitude of the pulse spike to be utilized for adjustment of the conductance change rate, that is, the learning rate in the hardware neural network system. Based on the TiO*_x_* memristor array, we design and implement compact convolutional neural network (CNN) hardware using multiple memristor arrays and a printed circuit board (PCB) to enable a fast‐converging in situ (FCIS) training that utilizes a high (low) learning rate in the initial (subsequent) iteration without any additional circuits. To verify the in situ training capability, binarized MNIST and 5‐class Clothes images are trained and evaluated. Compared to normal in situ training, the FCIS training effectively reduces the training iteration to half the initial loss function up to five times, indicating the fast‐converging feature in terms of the training cost. Consequently, it can achieve a maximum classification accuracy of ≈95.2% and ≈82.3% for the MNIST and Clothes image sets on a hardware implementation using FCIS training, offering the availability to utilize the stoichiometrically stabilized TiO*_x_* memristor array for the efficient neuromorphic computing hardware system.

## 2 Results and Discussions

### 2.1 TiO*_x_*‐Based 25 × 25 Memristor Array Device

**Figure 1:**
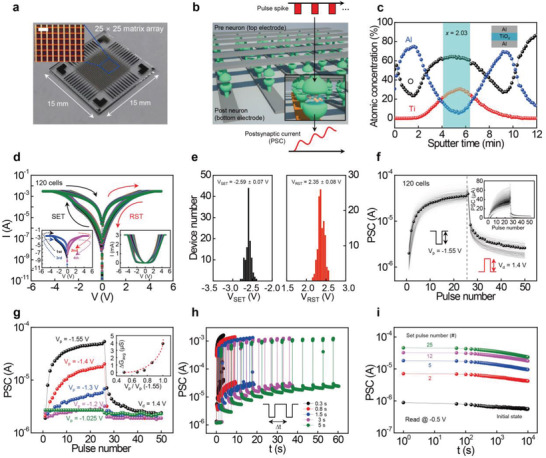
Device structure and switching characteristics of the TiO*_x_* memristor array. a) Optical microscopic and photographic images of a 25 × 25 passive matrix array TiO*_x_* memristor array with the scale bar of 500 µm. b) Schematic representation of a passive matrix array for an artificial neural network consisting of individual memristor cells between pre‐ and post‐neurons, generating PSCs driven by a presynaptic signal (pulse spike). c) XPS depth‐profiling analysis of the Al/TiO*_x_*/Al junction structure. The *x* value of the TiO*_x_* center region was measured as ≈2.03. d) The overlapping *I*–*V* curves of 120 selected TiO*_x_* memristor cells with the left and right inset showing the forming‐free resistive switching property (log(*I*) for *y*‐axis) and linear scale *I*–*V* plot, respectively. e) Statistical histogram of *V*_SET_ and *V*_RST_. f) The overlapping LTP/LTD characteristics of 120 TiO*_x_* memristor cells with *V*_p_ = −1.55 and *V*_d_ = 1.4 V. The number of programming pulses is 50. The PSC was read at −0.5 V. The inset shows the linear plot of the LTP/LTD curves. g) The LTP/LTD characteristics for different amplitudes of potentiating pulses ranging from −1.025 to −1.55 V at a fixed *V*_d_ = 1.4 V. The inset shows the plot of *∆G*_avg_ as a function of the relative *V*_p_ ratio. h) PSC responses driven by 12 repeated *V*_p_ = −1.55 V for 150 ms with different *∆t* ranging from 0.3 to 5 s. i) Retention test of PSC states triggered by different LTP steps from 2 to 25 spikes.

**Figure** **1**a shows an optical microscopic and a photographic image of a 25 × 25 passive crossbar array employing TiO*_x_*‐based memristor nodes. Each memristor cell is located in cross points of perpendicularly aligned Al electrode lines (width of 100 µm) possessing an Al/TiO*_x_*/Al layer structure on a glass substrate (15 mm × 15 mm). This crossbar array form can be utilized as the matrix constituent of an artificial neural network consisting of a two‐terminal artificial synapse linked between pre‐ and post‐synaptic neurons (Figure 1b). The postsynaptic current (PSC) of each memristor device cell is capable of gradual modification according to the history of applied pulse spikes from the presynaptic neuron, enabling learning and memory functions at a time.^[^ ^40^, ^41^, ^42^ ^]^ An investigation of the constituent elements in the TiO*_x_* memristor was performed by depth‐profiling X‐ray photoelectron spectroscopy analysis, as shown in Figure 1c. In previous studies, the importance of the oxygen concentration ratio (*x*) of a TiO*_x_* material has been emphasized because it definitely affects the resistive switching properties of the memory device with an oxygen vacancy (*V*_o_)‐based electrical conduction.^[^ ^43^, ^44^ ^]^ Accordingly, we designed a TiO*_x_* memristor presenting stable and reliable analog switching performances while securing a high device yield with optimal *x* value found to be ≈2.03, which is slightly higher than that of other typical TiO*_x_*‐based memories.^[^ ^45^, ^46^ ^]^ The effect of oxygen content variation is provided in Figure S1 and Note S1, Supporting Information, with regard to the investigation of electrical switching properties for various TiO*_x_* layer fabrication procedures in detail. Figure 1d shows the overlapping switching *I*–*V* curves of 120 randomly selected TiO*_x_* memristor cells in a 25 × 25 crossbar array. They all show an initial high resistance state (HRS) because the as‐fabricated device cells have the insulating Al–Ti–O layer at top interface, and exhibit a typical bipolar switching behavior operated by negative SET and positive RESET (RST) voltages without initial voltage forming process (Figure S1f,g, Supporting Information). In fact, the entire resistivity for device junction is determined at Al–Ti–O layer because the bulk resistance is much lower than the interface insulating region. When the negative voltage is applied to the top Al electrode, *V*_o_ can be generated by the diffusion of oxygen ions from the top Al/TiO*_x_* interface to the bulk active region, diminishing insulating nature of top interface and presenting a *V*_o_‐based conduction path between the top/bottom electrodes, that is, the SET process. However, as a positive voltage is applied, the oxygen ions start to move back to the top interface Al/TiO*_x_* region again, rupturing the conductive path and recovering the insulating nature of the top interface region, that is, the RST process.^[^ ^47^ ^]^ In this manner, the bipolar switching of the TiO*_x_* memristor device is deeply related to the reversible drift of oxygen ions between the top interface layer and oxygen‐deficient bulk region.^[^ ^48^, ^49^ ^]^ Notably, in our TiO*_x_* memristors, the entire switching *I*–*V* curves were observed without any switching failure, with the expectation of >99% device yield, which seems to almost coalesce as a single curve. Figure 1e shows the statistical distributions of *V*_SET_ and *V*_RST_ obtained by extrapolation in the linear region method^[^ ^50^ ^]^ for 120 TiO*_x_* memristor cells. In particular, the values for the mean and standard deviation of *V*_SET_ and *V*_RST_ are found to be −2.59 ± 0.07 and 2.35 ± 0.08 V, corresponding to variations of ≈2.7% and ≈3.4%, respectively. These low deviations of the operation voltages can result in uniform and reliable switching programming in the crossbar array structure. The statistical results for the device with lower junction area (four‐times) and array‐to‐array distribution with operational yield estimation are presented in Figure S2 and Note S2, Supporting Information. Because the TiO*_x_* memristive layer is deposited at entire array space without patterning, the device size is only determined by fabrication technique of top and bottom Al electrode lines, thus, it is expected to present reliable device performance in lower device dimension if more progressed electrode patterning process such as photolithography would be addressed in future work for the large‐scale device integration. Figure 1f shows gradual long‐term potentiation (LTP) and depression (LTD) of the overlapping PSCs for all 120 TiO*_x_* memristor cells according to the continuous potentiating and depressing input pulses (*V*_p_ = −1.55 V and *V*_d_ = 1.4 V for *t*_w_ = 150 ms) at a time period (*∆t*) of 800 ms. Notably, 120 randomly selected cells in the array show >99% working device yield not only for bipolar switching but also for analog synaptic functions as LTP/LTD with operational uniformity, indicating high reliability as neuromorphic device components. The process for obtaining analog switching LTP/LTD characteristics of the TiO*_x_* memristor device and its variation for different oxygen contents is presented in Figure S3 and Note S3, Supporting Information.

To control the degree of LTP change of the TiO*_x_* memristor cell, we applied different pulse trains consisting of five *V_p_* pulses for 150 ms in the range from −1.025 (green) to −1.55 V (black), as shown in Figure 1g. Note that the same pulse train consisting of *V*_d_ = 1.4 V for 150 ms is applied for the LTD process. Here, we observed that the average conductance change (Δ*G*_avg_), defined as (*G*_max_ − *G*_min_)/(pulse number), shows a nonlinear behavior, namely, presenting an abrupt change in the degree of PSC when *V*_p_ slightly varies (the inset of Figure 1g). This nonlinear pulse response can be utilized to efficiently modulate the conductance change rate even by the small range of voltages, which can adjust the learning rate in a hardware‐integrated neural network system (Note S4, Supporting Information).^[^ ^32^, ^33^, ^34^ ^]^ Figure 1h shows the PSC response of the TiO*_x_* memristor according to different *∆t* from 0.3 to 5 s (*V*_p_ = −1.55 V for 150 ms). The PSC change is larger for a shorter *∆t*, indicating frequency‐dependent synaptic plasticity. Additionally, each PSC exhibits a nonvolatile nature and nearly retains its own state for 10^4^ s (Figure 1i). Although there is a small decrease for each analog conductance state, the in situ training nature mitigates that variation by performing VMM inference with consistent conductance modulation for all synaptic weights during the training stage, resulting in no significant effect on hardware in situ training operation (Figure S23 and Note S8, Supporting Information). The DC sweep endurance and retention test were also evaluated, where an ON/OFF ratio of ≈5.74 × 10^1^ read at −0.5 V was well maintained during repeatable programming. In addition, the binary resistive switching property was also derived using the magnified pulse configuration as *V*_SET_ = −4 V with *t*_SET_ = 150 ms (Figure S4, Supporting Information). Note that the details of the LTP and LTD change under various operating voltage schemes with repetitive stability are presented in Figures S5‐1 and S5‐2, Supporting Information.

### 2.2 Repetitive Stability of the TiO*_x_* Memristor Cell

**Figure 2:**
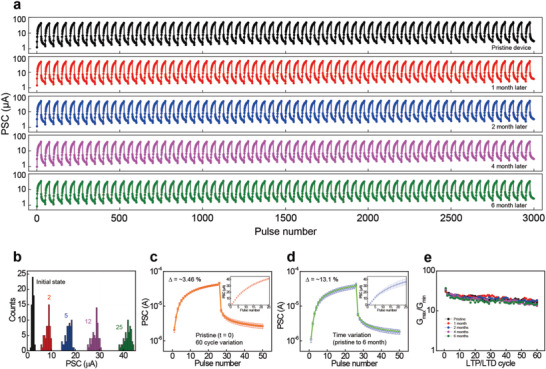
Stability of LTP/LTD characteristics of the TiO*_x_* memristor array. a) The whole repetitive LTP/LTD characteristic plots during a total of 3000 spikes of presynaptic pulses for the TiO*_x_* memristor cells under pristine conditions and after elapsed times (1, 2, 4, and 6 months). b) Statistical distribution of PSC levels during 60 LTP/LTD cycles. Each PSC level was measured at initial and different LTP steps from 2 to 25 pulses. The statistical LTP/LTD curves upon the variation of c) the pulse cycle and d) the elapsed time with the linear plots of the LTP region. e) The *G*_max_/*G*_min_ ratio as a function of LTP/LTD cycle for different elapsed times.

To implement practical and robust neuromorphic computing hardware, stable LTP and LTD functions of the memristor cell under repeated pulse cycles during long elapsed times are essential prerequisites.^[^ ^21^, ^22^ ^]^ **Figure** **2**a shows the repetitive transitions between the LTP and LTD functions during a total of 3000 presynaptic pulses (corresponding to 60 cycles) according to each elapsed time. Each pulse cycle consists of 25 potentiating pulses and 25 depressing pulses (*V*_p_ = −1.55 V, *t*_w_ = 150 ms, *V*_d_ = 1.4 V, and *∆t* = 800 ms). The stable LTP and LTD functions are well maintained not only under continuous input pulse stress but also after elapsed time under ambient conditions. Concretely, stable LTP and LTD functions are demonstrated during 6 months of elapsed time (black line for pristine device, red line for 1 month, blue line for 2 months, magenta line for 4 months, and green line for 6 months), indicating optimistic prospect for practical analog neuromorphic computing device application. In addition, each PSC level is reliable and maintained under repetitive pulse stress. Figure 2b shows the statistical distributions of the PSC levels during 60 pulse cycles for the pristine device, in which each PSC level was measured at the initial state and after different numbers of LTP steps (2, 5, 12, and 25). These well‐separated and uniform PSC levels imply that it can be reliably updated for the desired synaptic weight at an acceptable scale. Figure 2c,d shows the average LTP/LTD curves with a standard deviation of each PSC state during 60 pulse cycles for the pristine device (Figure 2c) and in the first cycle from pristine device to 6 months (Figure 2d). The overall statistics of the LTP/LTD curves are reliably conserved on a similar conductance scale. In particular, the standard deviation of each LTP/LTD curve is found to be ≈3.46% and ≈13.1% for the pulse cycle and time variation, respectively. These low deviations of the LTP/LTD function can enhance the analog computing performance, which is strongly dependent on the precision of the ensemble conductance map of memristor arrays.^[^ ^23^ ^]^ Figure 2e shows the evolution of the dynamic range, defined as the conductance ratio between the highest and lowest analog conductance states (*G*_max_/*G*_min_) of the TiO*_x_* memristor device, for different device elapsed times and pulse cycles. The effect of the device elapsed time and pulse stress on the LTP/LTD steps is negligible, indicating the excellent durability of the TiO*_x_* memristor device.

### 2.3 Hardware Integration Using the TiO*_x_* Memristor Arrays

**Figure 3:**
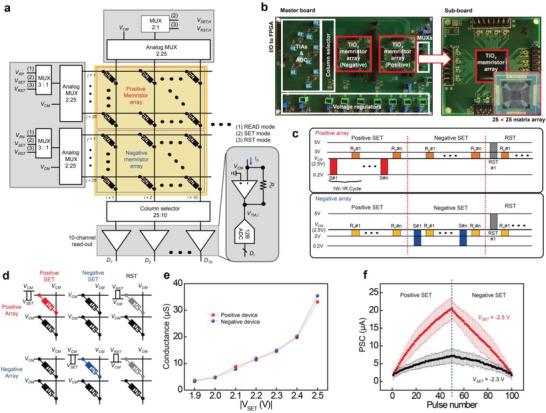
Hardware integration of TiO*_x_* memristor array and its operations. a) Schematic representation of the mixed signal interface between the two (positive and negative) 25 × 25 memristor arrays with the analog MUXs, TIAs, and ADC. The feedback resistance of the TIA is designed to be selected depending on the output current range. b) Optical image of the master and sub‐PCBs, including wire‐bonded memristor arrays and peripheral circuits. The columns of the memristor arrays can be connected to the input of the TIAs through the column selector. c) Pulses applied to the positive and negative array for positive SET (positive array, 500 ms, −2.5 V), negative SET (negative array, 500 ms, −2.5 V), and RST (both, 500 ms, 2.5 V). d) Voltage applying scheme to rows and columns for the positive SET, negative SET, and RST processes. e) The maximum conductance range of positive and negative arrays for the amplitude of the SET pulse. The conductance is driven by 50 repeated SET pulse trains with different *V*_SET_ values ranging from 1.9 to 2.5 V for the positive and negative memristor devices. f) Effective LTP/LTD functions triggered by positive and negative SET for *V*_SET_ = −2.5 and −2.3 V.

Using the implemented 25 × 25 TiO*_x_* memristor array device, a hardware system was integrated on a PCB with interface circuits to read and write the conductance of memristors (**Figure** **3**a). The PCB consists of a master board and two sub‐boards with wire‐bonded memristor arrays (Figure 3b). Each pair array comprises a positive and a negative memristor array, and the effective conductance values of the entire matrix can be determined by the conductance differences between positive and negative memristor arrays.^[^ ^27^, ^28^, ^29^ ^]^ To execute the VMM in the hardware system, the interface circuits surrounding the memristor devices were utilized as read‐out circuits, analog multiplexers (MUX), and voltage regulators. The read‐out circuit consists of 10‐channel transimpedance amplifiers (TIAs) and a 12‐bit analog‐to‐digital converter (ADC). The analog MUXs are designed to control the potential difference between each column and row of the memristor array depending on the location of the target cell and the three different operation modes, that is, READ, SET, and RST (Figure 3a and Figure S6, Supporting Information). The voltage regulators generate the reference voltages for the operation modes of the memristor device, that is, *V*_RP_ (0.5 V), *V*_RN_ (−0.5 V), *V*_SET_ (−2.5 V≈−2.3 V), *V*_RST_ (2.5 V), *V*_SET,H_ (−1.25 V), and *V*_RST,H_ (1.25 V) at *V*_CM_ (0 V). The read process for memristor arrays is carried out by applying input voltage to the rows of positive and negative arrays. Then, the output voltage of TIA (V_TIA,_ *_i_*) on the *i*th column in the array can be obtained with cumulative addition along the *j*th row, where *V*_TIA,_ *_i_* is determined by Equation (1):

(1) $$V_{\mathrm{TIA},i}=\left(\sum_{j=1}^{n}|G_{\mathrm{pos},i,j}|\cdot V_{P}\left(j\right)+\sum_{j=1}^{n}|G_{\mathrm{neg},i,j}|\cdot V_{N}\left(j\right)\right)\cdot R_{F}$$

where *G*_pos,_ *_i,j_* and *G*_neg,_ *_i,j_* are the conductance of the memristor on the *j*th row and *i*th column in both the positive array and negative array, respectively, *R*_F_ indicates the feedback resistor of the TIA, *V*_P_ (*j*) is a binary vector that can be *V*_RP_ or *V*_CM_ (*V*_N_ (*j*) = *V*_RN_ or *V*_CM_), and *n* is the number of rows in memristor array. Therefore, the device cells on the target row are connected to *V*_RP_ or *V*_RN_, while others are connected to *V*_CM_. Additionally, when *V*_RP_ or *V*_RN_ is applied to multiple rows, the VMM operations are carried out by summing the current from each device cell (Figure S7, Supporting Information). The current sum *I*_S_ is converted to *V*_TIA,_ *_i_*, depending on the *R*_F_ value. Each *V*_TIA,_ *_i_* is converted to digital signals *D_i_* by a 12‐bit ADC, which are then transmitted to the FPGA board (Note S5, Supporting Information).

The cell‐by‐cell write process of the memristor array based on the 1 write–1 read scheme^[^ ^35^, ^51^ ^]^ is useful to measure the device switching behavior and statistical characteristics. A complementary write scheme based on two devices (positive and negative) as one pair^[^ ^52^ ^]^ is employed to achieve a gradual conductance change as effective LTP and LTD operations. The write process is divided into the positive SET, negative SET, and RST schemes (Figure 3c,d). Here, in the measurement on the board, the writing condition is quite different from the condition before hardware integration as shown in Figure 1f,g. Although it is hard to clarify exactly the reason of the difference between them, it can be reasonably inferred that it is mainly due to the measurement environment change including increased resistance of line interconnection between the device array and board.^[^ ^53^, ^54^ ^]^ The proper conditions of writing pulse were experimentally found through the trials and error to obtain the optimal performance of the programming of the in situ training. During the positive SET, LTP writing and read pulses are sequentially applied to the target device (*j*th row) of the positive array, while only read pulses are applied to the corresponding device (*j*th row of the negative array). When read pulses are applied to both positive and negative devices, the effective conductance is shown as *G*_pos_ − *G*_neg_. As a result, the effective LTP function can be successfully mimicked because the conductance of the positive device cell (*G*_pos_) becomes relatively larger than that of the negative device cell (*G*_neg_). As more LTP pulses are applied to the positive device, the effective conductance increases gradually. During the negative SET, in contrast, LTP writing pulse is applied only to the target device cell of the negative array, which results in a reduction in the effective conductance (effective LTD function). These writing processes are reliably achieved on the target cells at the array level without the unwanted conductance change of other nontarget memristor cells due to the improved switching threshold uniformity (Figure S8, Supporting Information). Note that when the conductance of positive and negative cells is saturated in a low resistance state, the RST process with a 2.5 V amplitude is performed to change the conductance states to the HRS to reflect the synaptic weight beyond the conductance range of the memristor device (Figures S19 and S20, Supporting Information).

In addition to the switching behaviors, the statistical characteristics of the memristor arrays on the PCB should be evaluated before training for set‐up of the in situ training condition considering the surrounding factors of on‐board system. Figure 3e shows the conductance of representative memristor devices in positive and negative arrays after applying a 50 SET pulse train consisting of different magnitudes of *V*_SET_ from 1.9 to 2.5 V for 500 ms. We utilized only the LTP characteristics in a single memristor device for effective LTP (positive SET) and effective LTD (negative SET) functions of the pair array structure, resulting in an extremely low asymmetry value of ≈1.43,^[^ ^55^ ^]^ which is significantly improved compared to the asymmetry value of ≈43.41 in the single memristor device LTP/LTD curves (Figure 1f). This could lead the high performance of recognition for the hardware neuromorphic system. Meanwhile, due to the nonlinear pulse response of the TiO*_x_* memristor device (Figure 1g), which is the large variation of the conductance range for a small difference in *V*_SET_, the effective LTP/LTD functions can be uniformly adjusted over the whole array by fine‐tuning the amplitude of the SET pulse (Figure 3f). Differentiated with tuning the pulse for the individual device to improve the limitation of the device,^[^ ^56^ ^]^ the high uniformity of the response to the fine‐tuning makes this TiO*_x_* memristor be a candidate device for the optimized in situ training to control the learning rate of the training. Other statistical characteristics for conductance variations, ON/OFF ratio, and multilevel conductance states of positive and negative memristor arrays are presented in Figures S9 and S10, Supporting Information.

### 2.4 The CNN with FCIS Training Using the Integrated Circuits

**Figure 4:**
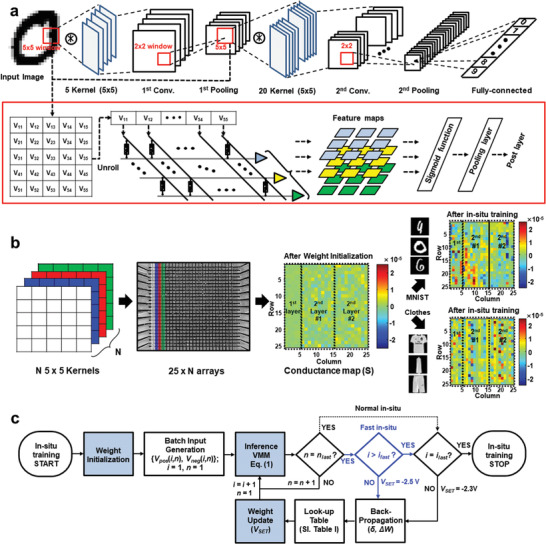
FCIS training of memristor‐based hybrid CNN hardware. a) Architecture of multiconvolution layers of CNN using memristor‐based VMM modules. The 28 × 28 images are applied to the convolution layer as a partial 5 × 5 images, which is unrolled as a 25‐length vector form, and one 5 × 5 kernel matrix is implemented by two columns with 25‐length (50 cells) of memristor arrays (positive and negative) connected in series. After the feature maps are produced through VMMs of input vectors and kernel conductance in convolution layer, they are fed forward as input of postlayers through the sigmoid activation function and max pooling layer. b) Implementation of kernel matrix with differential memristor array pairs and an analog conductance map before and after in situ training for two different image data sets (MNIST and Clothes). c) A flow diagram of the in situ training for 4000 iterations. The variables *i* and *n* represent the index of the iteration and the sample image in the batch, respectively. The highlighted path represents the FCIS training, while the dashed path represents the normal in situ training.

Using a PCB (Figure 3b) as a single VMM module, multiconvolution layers of the CNN for 28 × 28 binary MNIST patterns are constructed by three VMM modules (**Figure** **4**a and Figure S11, Supporting Information). The CNN is based on the LeNet model with two convolution and pooling layers and one fully connected (FC) layer.^[^ ^57^ ^]^ The first and second convolution layers, which consist of five 5 × 5 kernel matrices and twenty 5 × 5 kernel matrices, are implemented by three VMM modules. The rest of the CNN, including the first and second pooling layers and FC layer, is implemented in the software (Figure S13‐2, Supporting Information). The CNN is trained for 4000 iterations among the 60 000 and 30 000 images of MNIST^[^ ^57^ ^]^ and the five‐class Clothes training data set^[^ ^58^ ^]^ (Figure S12 and Note S6, Supporting Information), respectively, with a batch size of 50. For the in situ training process, first, the RST process is performed to initialize the conductance of the entire memristor array in the HRS (weight initialization), and then the conductance maps for each kernel matrix are properly updated along the classified image data set (Figure 4b). The classification accuracy is evaluated after every 400 iterations using 3000 test images of each data set. Details of the overall procedure for the in situ training of the CNN are presented in the flow diagram of Figure 4c.

Before the training, weight initialization is performed; then, the binary input vectors are generated from the image data set and applied to the network array during the inference phase (Figures S13‐1 and S14a, Supporting Information). After the initial inference through all layers, the software‐implemented backpropagation algorithm proceeds to determine the amounts of the weight update, Δ*W*, for all kernels. Then, all values of Δ*W* are converted to the number of required writing pulses to be applied to each cell in the array according to the look‐up table (LUT, Table S1, Supporting Information). Consequently, the converted numbers of pulses apply to the corresponding rows of the memristor arrays simultaneously, and the conductance is updated in column‐by‐column (Figure S14c, Supporting Information). In FCIS training, notably, we set the conductance to be updated in two steps: *V*_SET_ = −2.5 V in the initial iterations for fast convergence and *V*_SET_ = −2.3 V after the 500th iteration. On the other hand, *V*_SET_ is constantly maintained at −2.3 V in the case of normal in situ training. Note that a flow diagram and conductance map of the hybrid training are shown in Figure S15, Supporting Information.

**Figure 5:**
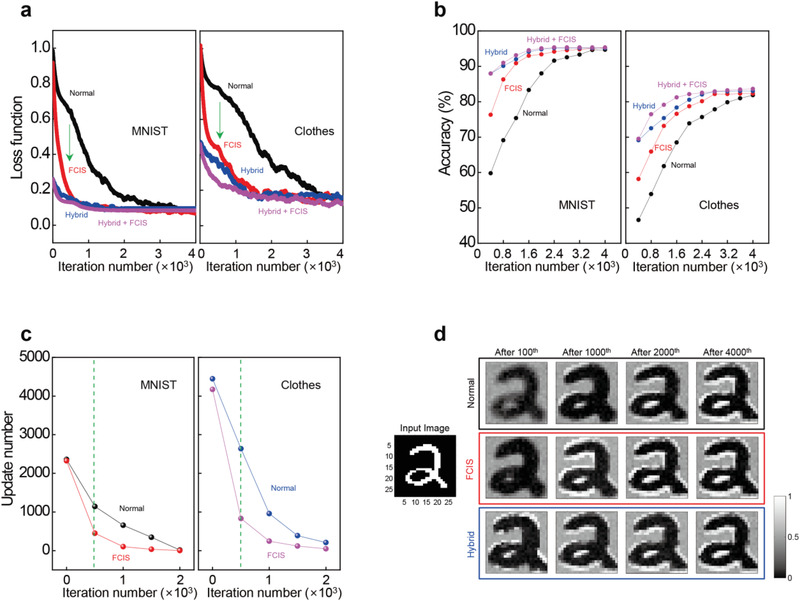
Classification performance comparison for the four different in situ training strategies. a) Normalized loss function of the CNN for normal, FCIS, hybrid, and hybrid with FCIS training. b) Classification accuracy of the CNN for normal, FCIS, hybrid, and hybrid with FCIS training. c) The total number of required training pulses at the initial, 500th, 1000th, 1500th, and 2000th iterations, which is calculated and converted from the backpropagation algorithm and look‐up table (Table S1 and Figure S16, Supporting Information). d) The 24 × 24 output feature maps of the first convolution layer, for example, image “2” during FCIS, normal, and hybrid training.

As shown in **Figure** **5**a, fast convergence in FCIS training can definitely be observed compared with the normal in situ training until the 500th iteration for both the MNIST and Clothes image sets. The values of the normalized loss function at the 500th iteration for the MNIST data set are 0.63 and 0.17 for normal in situ and FCIS training, respectively, and in the case of the Clothes data set, they are 0.78 and 0.45. Similarly, while it is less effective than normal training, the hybrid training with FCIS training demonstrates efficient convergence in several initial iterations than only hybrid training, despite the preprogramming of transfer learning that has an effect of reducing the initial loss function. In both hybrid training cases, the initial loss functions start from 0.26 and 0.46 instead of 1 for MNIST and Clothes data, respectively, due to the preprogramming. Comparing the loss function at 500th iteration, it is found that FCIS training is more effective for the Clothes data than MNIST in that the differences of the loss functions between the hybrid with FCIS training and only hybrid training is 0.024 and 0.104 for MNIST and Clothes data, respectively. In addition, comparing the number of iterations required to reach half the initial loss function highlights the efficiency of the FCIS training in that the normal in situ and FCIS training records 741 and 147 for MNIST and 1520 and 296 for the Clothes data set, respectively, which indicates greater than five‐times‐faster convergence of the FCIS training. The rapid convergence of the FCIS is because the large amount of the weight update is required at the initial training stage, where the weight is updated from the initial state as shown in Figure 4b. The relatively large learning rate up to 500th iteration of FCIS training can reduce the loss function faster than the fixed learning rate in the normal in situ training. Meanwhile, since the large learning rate disturbs the fine convergence of the network, the reduced learning rate after 500th iteration is applied for the loss function to be converged to the saturation point.

Figure 5b shows the evolution of the classification accuracy over the training iterations for the four different training methods. While classification accuracies of 97.3% and 85.2% are achieved in the software simulation for the MNIST and Clothes images, respectively, those of the hardware in situ training reaches 94.2% and 81.9% despite the existence of the nonideal characteristics of the memristor. Notably, this outcome shows that the FCIS training rapidly increases the classification accuracy to 95.2% and 82.3% compared to the normal in situ training, and FCIS training shows higher classification accuracy as 95.3% and 83.6%, respectively, even with the initial transfer learning. Here, the testing process to verify the accuracy immediately started as soon as one training iteration ends without any time delay as the in situ training perspective. In addition, FCIS training can significantly reduce the total number of required updating pulses (Figure 5c), which results in enhancement of the energy efficiency for the training process (Figure S21 and Note S7, Supporting Information). The total numbers of writing pulses in FCIS training steeply decrease from 2325 to 450 for MNIST images and from 4170 to 830 for Clothes images until the 500th iteration and then slightly decrease after the 1000th iteration. The usefulness of FCIS training can be clearly revealed through the output feature maps output during the training. Figure 5d shows transitions of the output feature maps of the first convolution layer, for example, image “2” for three different training schemes according to the number of training iterations. As the iteration progresses, the feature map of FCIS training changes roughly but rapidly until the 1000th iteration due to the high rate of conductance change, while a steady transition is shown in the normal in situ training (Video S1, Supporting Information). Representative examples of intermediate feature maps of the first and second convolution layers are shown in Figure S17, Supporting Information, and further training results using short pulse width are provided in Figure S18 and Table S2, Supporting Information. The effect of memristor device nonidealities such as conductance variation with programming error, stuck‐ON, and stuck‐OFF on in situ training performance is presented in Figure S22, Supporting Information. In addition, we suggested a more sophisticated training algorithm of RMSprop to adjust the learning rate in the hardware level by using pulse amplitude tuning as well as the number of pulses in Figures S24 and S25 and Note S9, Supporting Information.

## 3 Conclusions

We constructed the CNN hardware using multiple TiO*_x_* memristor arrays equipped with high operational uniformity and reliability in the synaptic functions for efficient in situ training. The TiO*_x_* memristor was fabricated using stoichiometrically stabilized TiO*_x_* material and integrated on a PCB unit as a 25 × 25 array, achieving >99% operational yield, outstanding uniformity of the switching threshold with relative deviation for up to ≈2.7%, extremely low asymmetry value of ≈1.43, and high functional stability with an over 3000 programming cycle repeatability and 6 months of device elapsed time (Tables S3‐1 and S3‐2, Supporting Information). In addition, the nonlinear PSC response allowed adjusting the conductance change rate by fine‐tuning the pulse amplitude, thus modulating the learning rate during the training process. With the reliable and superior symmetrical analog switching characteristics of the TiO*_x_* memristor pair array, the implemented CNN hardware offers reliable programming procedures and FCIS training through adjustment of the learning rate. Four different training strategies, that is, FCIS, normal, hybrid training, and hybrid with FCIS training, for the MNIST and Clothes images were designed and evaluated. Notably, FCIS training can significantly reduce the training cost about five‐times compared to normal in situ training without periphery circuit for individual voltage tuning at ≈95.2% and ≈82.3% accuracy for the MNIST and Clothes image sets, respectively. This study suggests the realization of memristor‐based neuromorphic computing system using passive array memristor operation to represent the global constant programming voltage scheme for entire cells and learning rate modulation according to training stage for fast and energy efficient artificial neural network application.

## 4 Experimental Section

### Fabrication Process of the TiO*_x_* Memristor Array Device

The bottom Al electrode, as a postsynaptic neuron, was deposited at a thickness of 30 nm on a bare glass substrate (15 × 15 mm^2^) by thermal evaporation after substrate cleaning by acetone and isopropyl alcohol in an ultrasonication bath for 10 min. The TiO*_x_* memristive layer was formed directly on the bottom electrode to a thickness of 20 nm by a radio frequency magnetron sputtering process at a base pressure of ≈8.0 × 10^−6^ Torr and working pressure of 5.0 mTorr using a TiO*_x_* target (rutile, 99.95% purity) with a sputtering power of 175 W for 10 min. The top Al electrode, as a presynaptic neuron, was deposited at a thickness of 30 nm perpendicular to the line direction of the bottom electrode, presenting 25 × 25 crossbar array memristor devices with a cell junction size of 100 × 100 µm^2^. For circuit integration on the PCB unit, the electrical pad of the array device was linked to those of the PCB using a conventional wire‐bonding process to enable the programming of the individual memristor array cells for hardware implementation.

### Electrical Measurement of the TiO*_x_* Memristor Array Device

To characterize the electrical properties of the TiO*_x_* memristor array devices, an Agilent 4155C semiconductor parameter analyzer and 81104A pulse generator were used, which were connected to an ambient probe station. For the evaluation of *I*–*V* characteristics of the TiO*_x_* memristor in device level, the voltage was applied at top Al electrode maintaining bottom Al electrode grounded (word/bit access for the cross‐point at passive scheme). The other word/bit lines except the target cell were kept in a floated without any bias access.

### Inference of the CNN

During the inference for one sample image, total 896 times of analog VMMs were performed by applying the 576 to the one VMM module and 320 input vectors to the two VMM modules for the first and second convolution layers (Figures S13‐1 and S13‐2, Supporting Information). The 25‐dimensional input vectors were fed forward to each convolution layer by applying a read voltage (*V*_RP_ or *V*_RN_) and *V*_CM_ to the rows of the memristor array as binary 1 and 0 that did not require the read voltage amplitude encoding. Then, the ADC converted the output voltages of the TIAs after 10 µs. It took 28 µs for one VMM operation to be completed, including the time needed for serial communication between MUXs and the ADC. After the output digital signal was transmitted to the PC, the ADC output of each channel was applied to the modified sigmoid function as follows in Equation (2):

(2) $$y\left(v\right)=\frac{1}{1+e^{-g\cdot (v-a)}}$$

where *v*, *g*, and *a* were the TIA output voltage in Equation (1), gain, and offset of the input voltage, respectively. The values of *g* and *a* were set as (1, 2.5) and (2, 2.5) in the first and second layers, respectively, considering the different numbers of connections at each layer. The 24 × 24 and 8 × 8 matrices of the sigmoid output in the first and second layers were downsampled to 12 × 12 and 4 × 4 using 2 × 2 filters in the pooling layers. Since the number of output feature maps in the second pooling layer was 20, a 320‐dimensional vector was fed into the FC layer in the digital computer. For consistency with hardware, the weights and neurons in FC layer were encoded to have a same conductance (2∼30 µS), voltage range (0∼5 V), and resolution of the memristor (analog 32‐states, equally 5‐bit in digital domain) and read‐out circuits (12‐bit) based on the measurement data. After inference, the loss function for the *i*th iteration (*C_i_*) was calculated by the addition of the (*i*−*1*)th loss function and the current mean square error (*E_i_*) as follows in Equations (3) and (4).

(3) $$C_{i+1}=\left(1-p\right)\cdot C_{i}+p\cdot E_{i+1}$$

(4) $$E_{i}=\frac{1}{2N}\cdot \sum_{n=1}^{N}{\left(y_{n}-t_{n}\right)}^{2}$$

where *y_n_*, *t_n_*, *N*, and *p* were the output of the *n*th neuron in the FC layer, the target output, the number of neurons in the FC layer, and the update constant (0.01), respectively.

### Applying the Backpropagation Algorithm

During the training phase, the amounts of weight updates Δ*W* between *i*th neuron at prelayer (*I*) and *j*th neuron at postlayer (*J*) were determined by the software‐based backpropagation algorithm in Equation (5).

(5) $$\Delta W_{ij}=\eta \cdot \delta_{j}\left(n\right)\cdot o_{i}\left(n\right)$$

where *η*, *o*, and *δ* were the learning rate, the feed‐forward output of neuron, and the back‐propagated error from the postlayer, respectively. The delta function *δ* was defined in Equation (6).

(6) $$\delta_{j}=\left\{\begin{array}{c} \left(o_{j}-t_{j}\right)\cdot o_{j}\cdot \left(1-o_{j}\right),J=L;\\ \left(\sum_{k\in K}w_{jk}\cdot \delta_{k}\right)\cdot o_{j}\cdot \left(1-o_{j}\right),\begin{array}{cc} & 0<J<L \end{array}. \end{array}\right.$$

where *k* and *l* represented the index of the neurons at the next‐layer after postlayer (*K*) and the last layer (*L*), respectively, and the capital letters represented the index of the layer. Here, the learning rate *η* in Equation (5) was set to 1 × 10^−7^. Based on the hardware scheme of the backpropagation using the convolution module in Figures S26‐1, S26‐2, and S27, and Note S10, Supporting Information, the computed values of Δ*W* were converted to the number of pulses to be applied to each cell through the look‐up table (Table S1, Supporting Information).

## Conflict of Interest

The authors declare no conflict of interest.

## Supporting information

Supporting Information

Supplemental Video 1

## Data Availability

The data that support the plots within this paper and other findings of this study are available from the corresponding authors upon reasonable request.
