# Supplementary material for: A Learning‐Rate Modulable and Reliable TiO x Memristor Array for Robust, Fast, and Accurate Neuromorphic Computing
Source: Adv Sci (Weinh). 2022 Jun 5;9(22):2201117. doi: 10.1002/advs.202201117 (PMC9353447; doi:10.1002/advs.202201117)
Supplement: Supplementary file 1 — Supporting Information [file ADVS-9-2201117-s002.pdf]

ADVANCED SCIENCE
Open Access

Supporting Information

for *Adv. Sci.*, DOI 10.1002/advs.202201117

A Learning-Rate Modulable and Reliable TiO_x Memristor Array for Robust, Fast, and Accurate Neuromorphic Computing

Jingon Jang, Sanggyun Gi, Injune Yeo, Sanghyeon Choi, Seonghoon Jang, Seonggil Ham, Byunggeun Lee and Gunuk Wang**

Copyright WILEY-VCH Verlag GmbH & Co. KGaA, 69469 Weinheim, Germany, 2016.

Supporting Information

A learning-rate modulable and reliable TiO_x memristor array for robust, fast, and accurate neuromorphic computing

Jingon Jang[†], Sanggyun Gi[†], Injune Yeo, Sanghyeon Choi, Seonghoon Jang, Seonggil Ham, Byunggeun Lee* and Gunuk Wang*

[†]These authors contributed equally to this work.

J. Jang[†], S. Choi, S. Jang, S. Ham, G. Wang*

KU-KIST Graduate School of Converging Science and Technology, Korea University, 145, Anam-ro, Seongbuk-gu, Seoul 02841, Republic of Korea

Gunuk Wang; Corresponding E-mail: gunukwang@korea.ac.kr

S. Gi[†], I. Yeo, B. Lee*

School of Electrical Engineering and Computer Science, Gwangju Institute of Science and Technology, 123, Cheomdangwagi-ro, Buk-gu, Gwangju, Republic of Korea

Byunggeun Lee; Corresponding E-mail: bglee@gist.ac.kr

G. Wang*

Department of Integrative Energy Engineering, Korea University, 145, Anam-ro, Seongbuk-gu, Seoul 02841, Republic of Korea

Keywords: Memristors, Artificial synapses, Neuromorphic computing, Uniformity, Hardware implementation

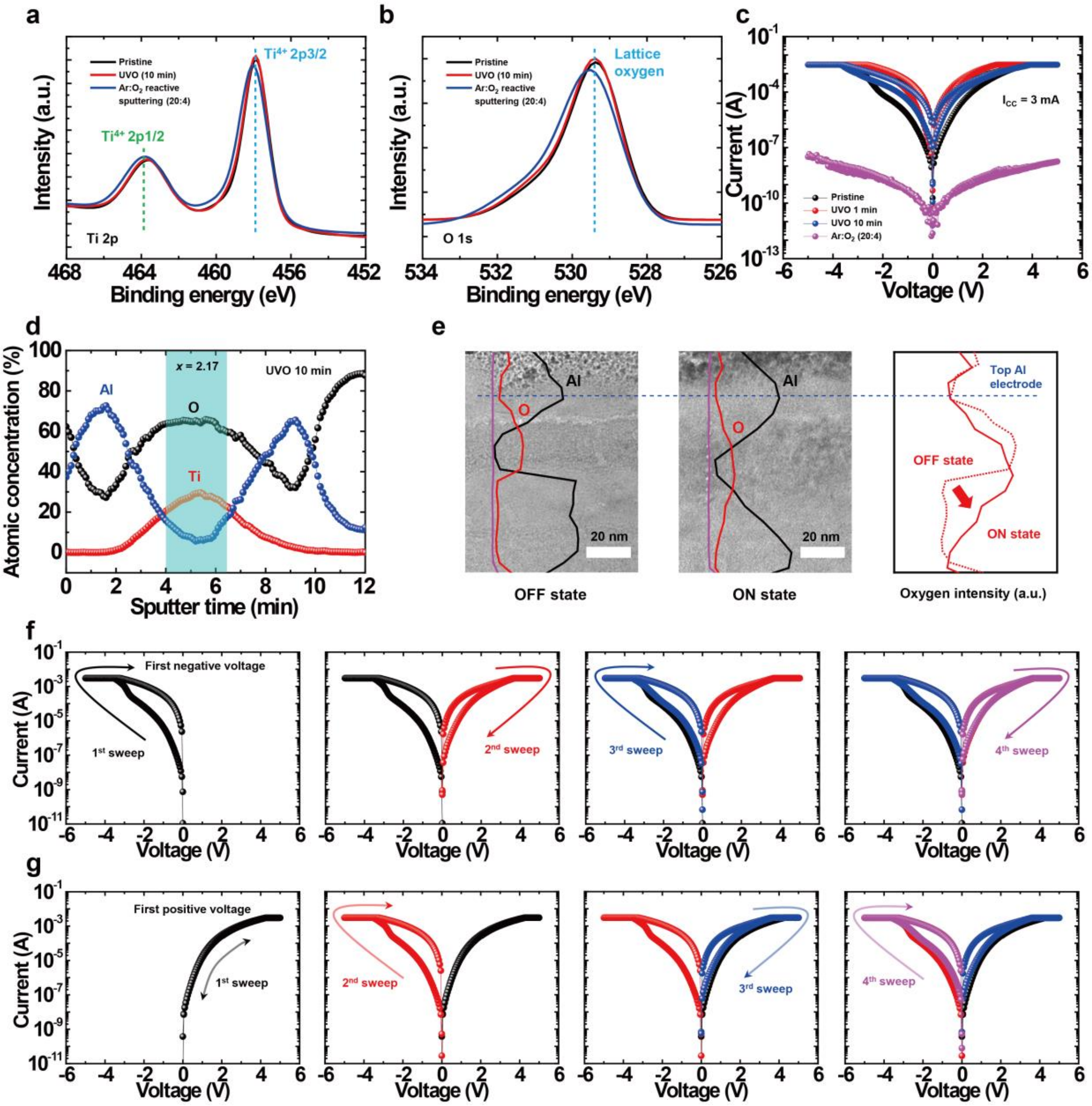

Figure S1. Effect of subsequent oxygen treatment on the TiO_x layer. XPS spectra of a) Ti 2p and b) O 1s for the TiO_x layer under pristine conditions (black line), with UVO treatment (red line), and under the reactive sputtering process by $Ar:O_2$ (20:4) (blue line). c) *I*–*V* curves of the TiO_x memristor device according to subsequent oxygen treatments. d) XPS depth-profiling analysis of the Al/TiO_x/Al junction structure after 10 min of UVO treatment. Note that the x value of the TiO_x center region is ~2.17 (see Supporting Note 1). e) The TEM analysis for each OFF (left plot) and ON state (middle plot) of the device, and relative oxygen intensity for each state (right plot). The *I*-*V* switching curve of pristine device at first f) negative and g) positive voltage sweep sequence, representing the forming-free switching behavior regardless of the programming scheme. Even in first positive voltage sweep in g),

the SET process occurred only in the negative voltage region (red and magenta line) while positive voltage sweep (black line) at HRS state did not make any change.

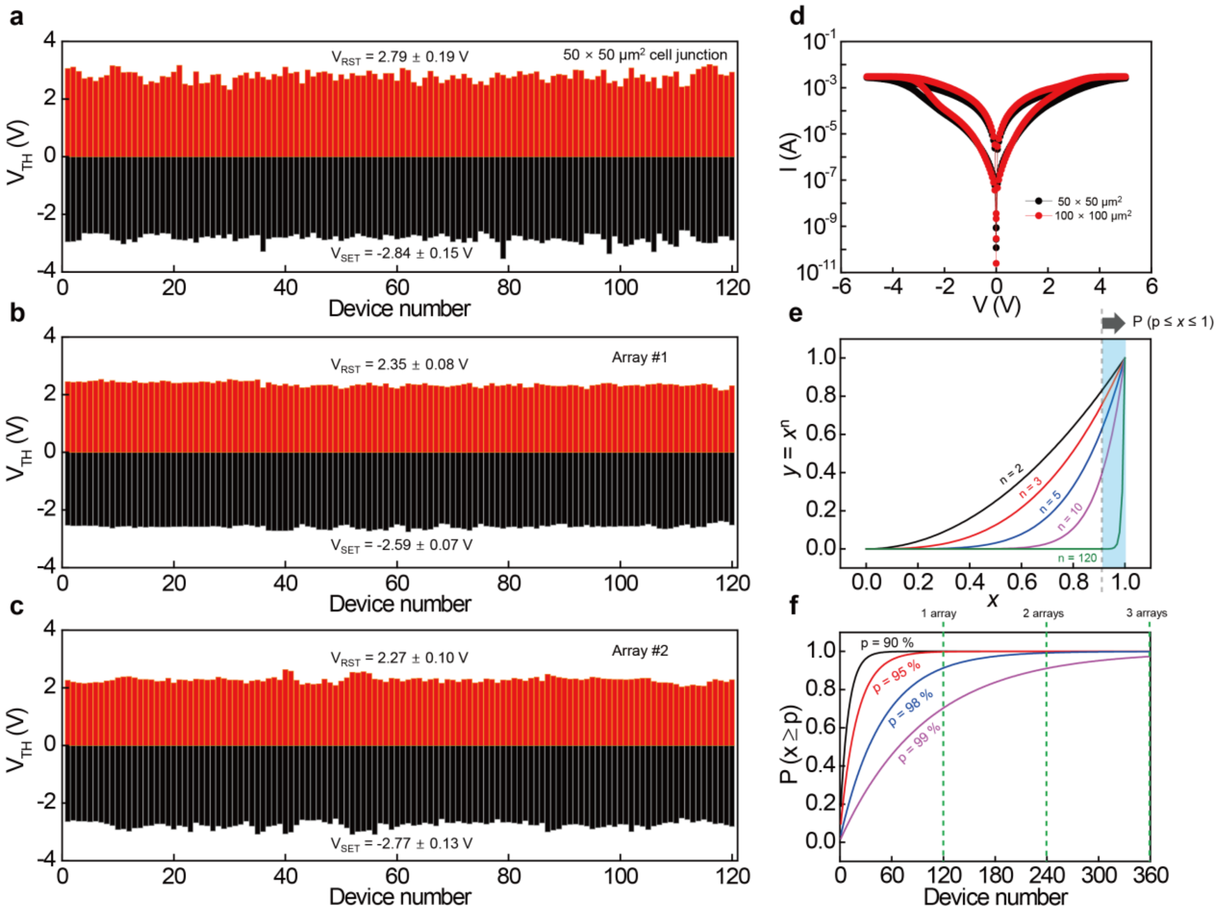

Figure S2. The statistical results of V_{SET} and V_{RST} for the device a) with 50 × 50 μm^2 of junction area and b,c) array-to-array variation. d) The *I*-*V* curves for a different cell junction area. The current ratio between 50 × 50 and 100 × 100 μm^2 device cell was obtained as ~1.64 (at –0.5 V), representing only little difference according to device size and the V_o filament switching formed at the top Al/TiO_x interface. e) The probability density function with different number of evaluated cells and f) the estimation of device operational yield. For the device with 50 × 50 μm^2 of the cell junction area, the bipolar resistive switching behavior and threshold voltage uniformity were observed in the same manner for the 100 × 100 μm^2 device because the vertical direction switching mechanism for Al/TiO_x/Al device junction is not influenced by the lateral device size defined as an overlapped region by top and bottom Al electrode lines except the quantitative amount of overall current level. For example, the V_{SET} and V_{RST} show -2.84 ± 0.15 and 2.79 ± 0.19 V for 120 devices, respectively, which indicates only little difference compared to original device (V_{SET} = -2.59 ± 0.07 and V_{RST} = 2.35 ± 0.08

V for 100 × 100 μm^2 devices). Consequently, the size of memristor junction does not affect the switching performance such as switching uniformity and conductance level.

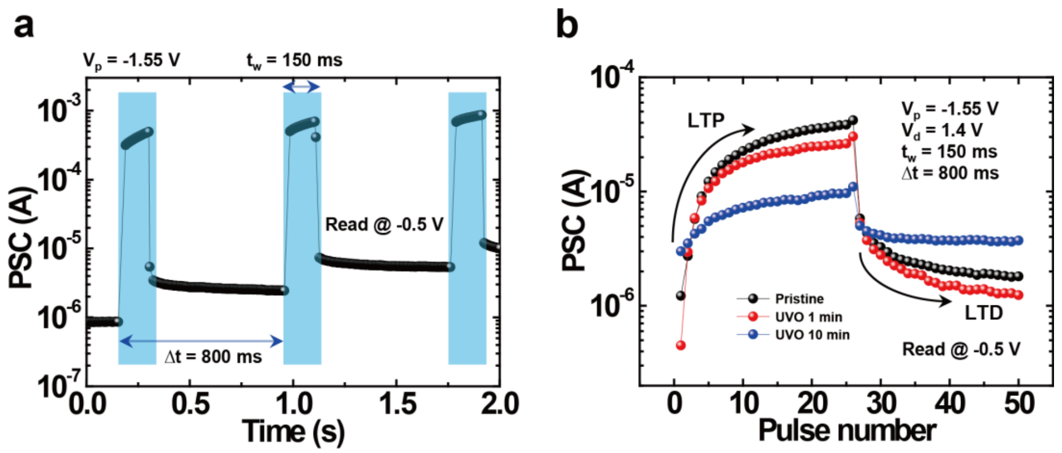

Figure S3. PSC response of the TiO_x memristor device. a) PSC response of the TiO_x memristor device at the presynaptic potentiation pulse (V_p = –1.55 V, t_w = 150 ms, and Δt = 800 ms). b) LTP/LTD curves of PSC as a function of the pulse number for different UVO treatment times (black line for pristine, red line for 1 min, and blue line for 10 min).

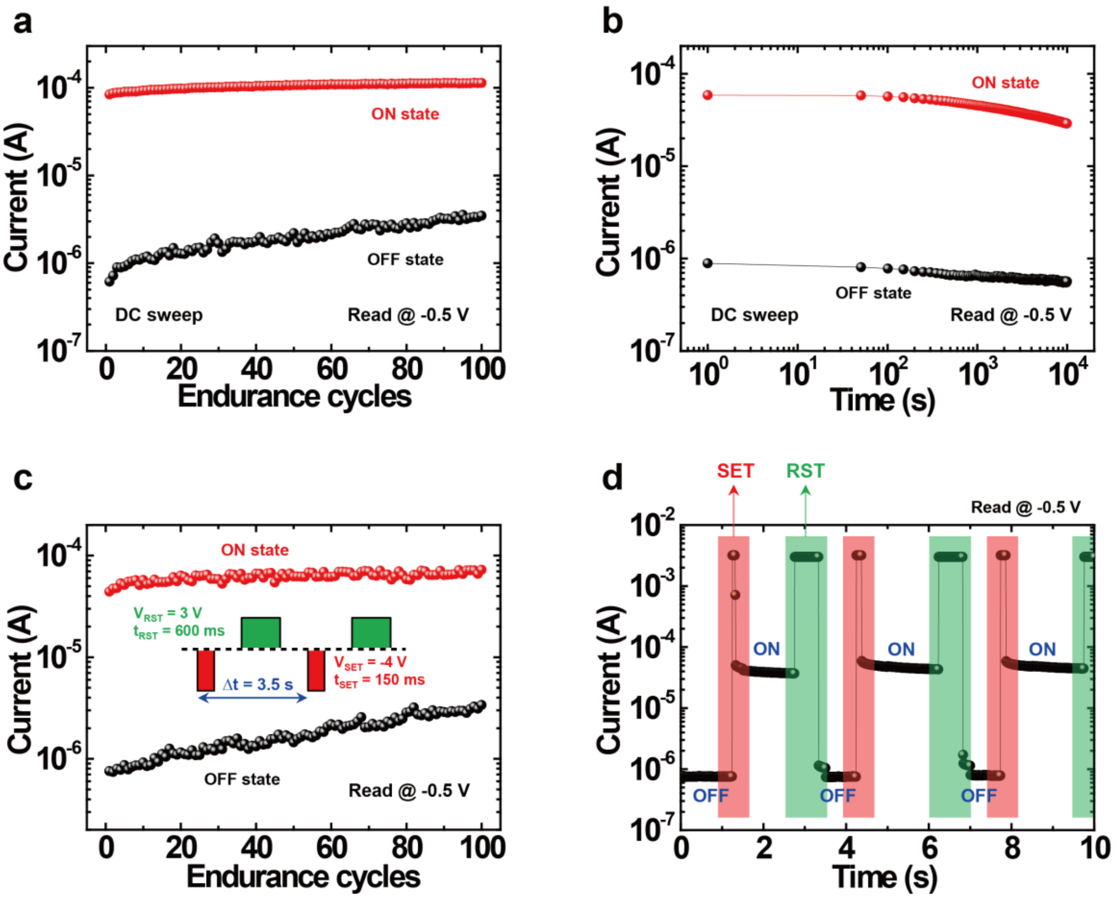

Figure S4. Resistive switching properties of the TiO_x memristor device. a) DC sweep endurance test of the TiO_x memristor device during 100 voltage sweep cycles, where the average ON/OFF ratio was found to be ~5.74 × 10^1 at a read voltage of –0.5 V, exhibiting stable binary operations. b) Retention property of each ON and OFF state for 10^4 s with 50 s intervals at a read voltage of –0.5 V. c) Endurance test over 100 cycles in pulse steps (V_{SET} = –4 V, t_{SET} = 150 ms, V_{RST} = 3 V, t_{RST} = 600 ms with Δt = 3.5 s). The red and green boxes represent the SET and RESET (RST) pulse trains, respectively. d) The current-time curves of the first few pulse periods in (c). The SET process (red box) converted the resistive state from OFF to ON, and the RST process (green box) converted the state from ON to OFF.

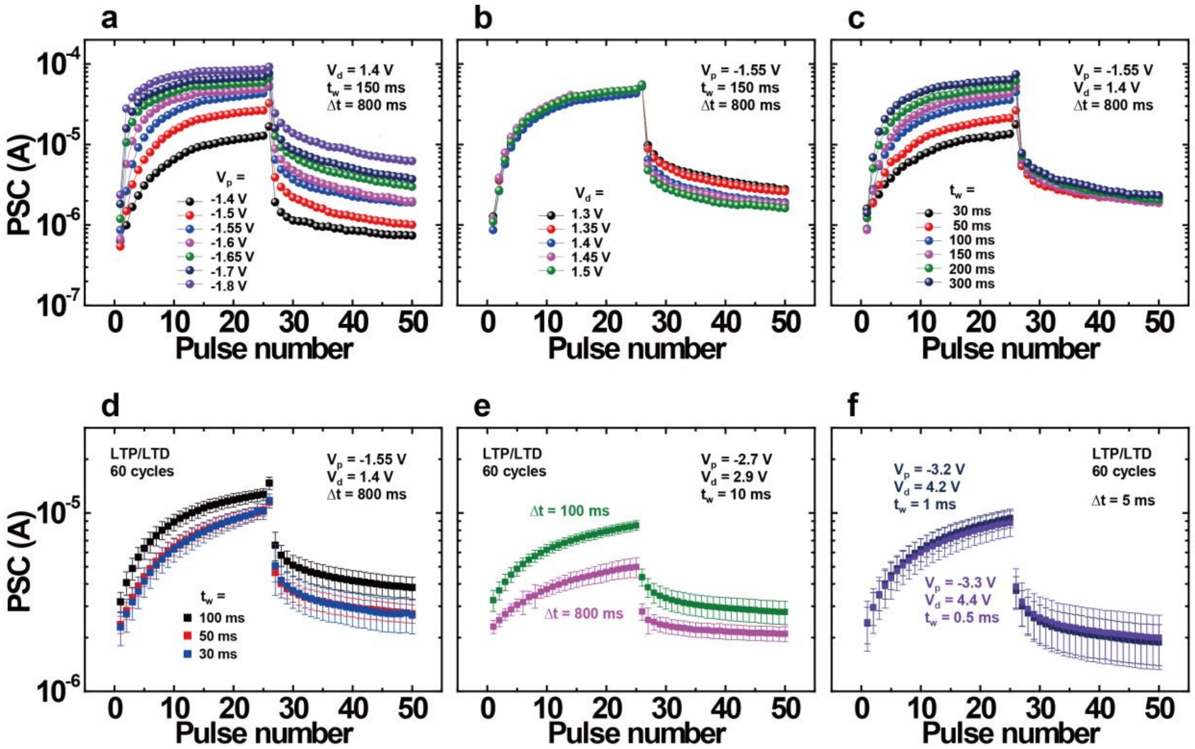

Figure S5-1. LTP/LTD curves of the PSC for different presynaptic pulse configurations. PSC responses of the TiO_x memristor device for different pulse amplitudes of a) the potentiation voltage (V_p), b) the depression voltage (V_d), and c) the pulse width (t_w). d-f) The statistical LTP/LTD curves during 60 pulse cycles. As the magnitude of potentiation pulses (V_p and t_w) increased, the PSC change increased at the same pulse number. Likewise, as the magnitude of V_d increased, the PSC decreased, but no significant variation was detected above 1.4 V. Note that the relative difference in potentiation voltage magnitude (Figure S5-1a) to obtain PSC

change is remarkably lower than that in pulse width (Figure S5-1c), indicating energy efficiency of programming pulse amplitude modulation for learning rate adjustment.

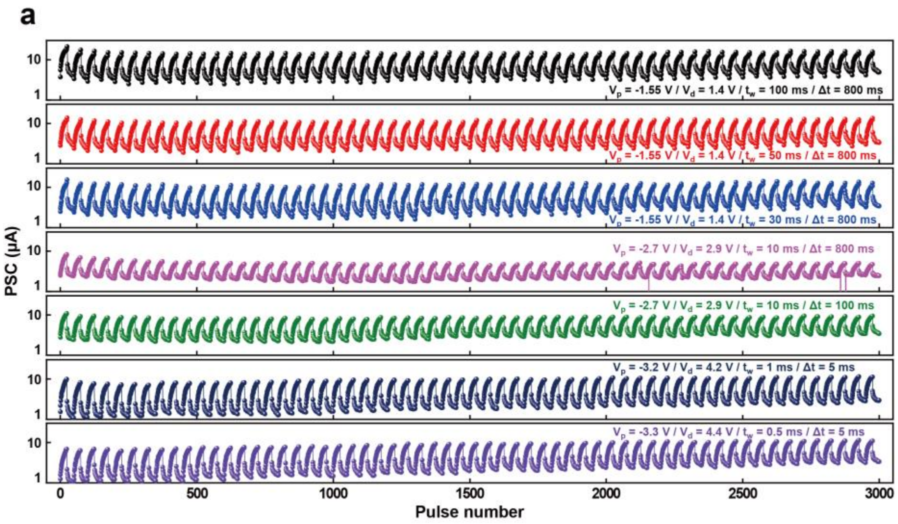

b

V_p (V)	V_d (V)	t_w (ms)	Δt (ms)	Error (%)
-1.55	1.4	150	800	3.46
-1.55	1.4	100	800	7.68
-1.55	1.4	50	800	7.63
-1.55	1.4	30	800	10.0
-2.7	2.9	10	800	12.8
-2.7	2.9	10	100	6.15
-3.2	4.2	1	5	11.9
-3.3	4.4	0.5	5	16.3

Figure S5-2. a) The whole repetitive LTP/LTD characteristic plots and b) error value as PSC variation during a total of 3,000 spikes of presynaptic pulses for different pulse parameters. When the pulse width was decreased with fixed other parameters (V_p = –1.55 V, V_d = 1.4 V, and Δt = 800 ms), the statistical parameter got worse where error value was ~7.68 % for 100 ms (black line), ~7.63 % for 50 ms (red line), and 10.0 % for 30 ms (blue line). However, by using the modulation of the V_p and Δt, it is also possible to reduce the pulse width with considerably maintaining (or weakening) PSC error value. In case of 10 ms of pulse width, the reliable LTP/LTD curves were obtained at -2.7 V of V_p, and the error showed ~12.8 % for 800 ms of the pulse period (magenta line). But when the pulse period was reduced to 100 ms, the error value was also reduced as ~6.15 % (green line) which is relevant to frequency-dependent synaptic plasticity in Figure 1h. Consequently, although there is a tradeoff issue between switching speed and uniformity, the reduction of statistical uniformity can be considerably mitigated by using modulation of pulse programming scheme (magnitude or period) in terms of frequency-dependent synaptic plasticity for practical application.

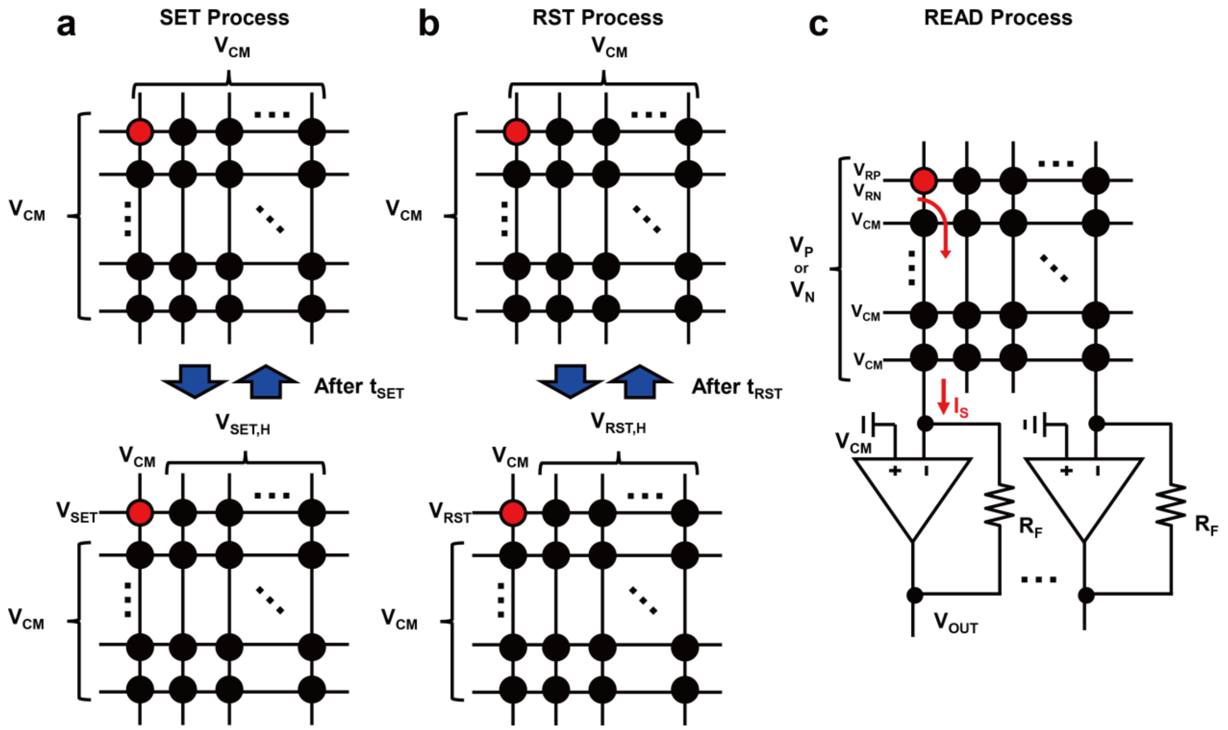

Figure S6. Dependence of the voltage applying scheme on the SET, RST, and READ processes. a) SET process. b) RST process. c) READ process. Initially, all rows and columns are connected to V_{CM}. When the device on the first row and column is under the SET (RST) process, the first row is connected to V_{SET} (V_{RST}), and all columns except the first one are connected to $V_{SET,H}$ ($V_{RST,H}$). After t_{SET} (t_{RST}) seconds, all columns and rows are connected to V_{CM} again. To read the conductance of the target device highlighted in red, the first row is connected to V_{RN} or V_{RP} depending on the polarity of the array, while the columns are connected to the input node of the TIAs, which is a virtual ground. Each reference voltage is actually implemented on a 2.5 V V_{CM} basis, which is described as 0 V in the main text for convenience. The R_F is determined by considering the current from the memristors and the output swing range of the TIA ([0 V, 5 V]) during the programming process for 1 device and the inference process for 50 devices. When the system is under the programming process for 1 device, the value of R_F is 250 KΩ because the maximum conductance range in the LRS per device is approximately 20 μS. On the other hand, when the system is under the inference phase, in which the current is generated from multiple devices, the value of R_F is determined by 10 KΩ, considering the worst-case pattern.

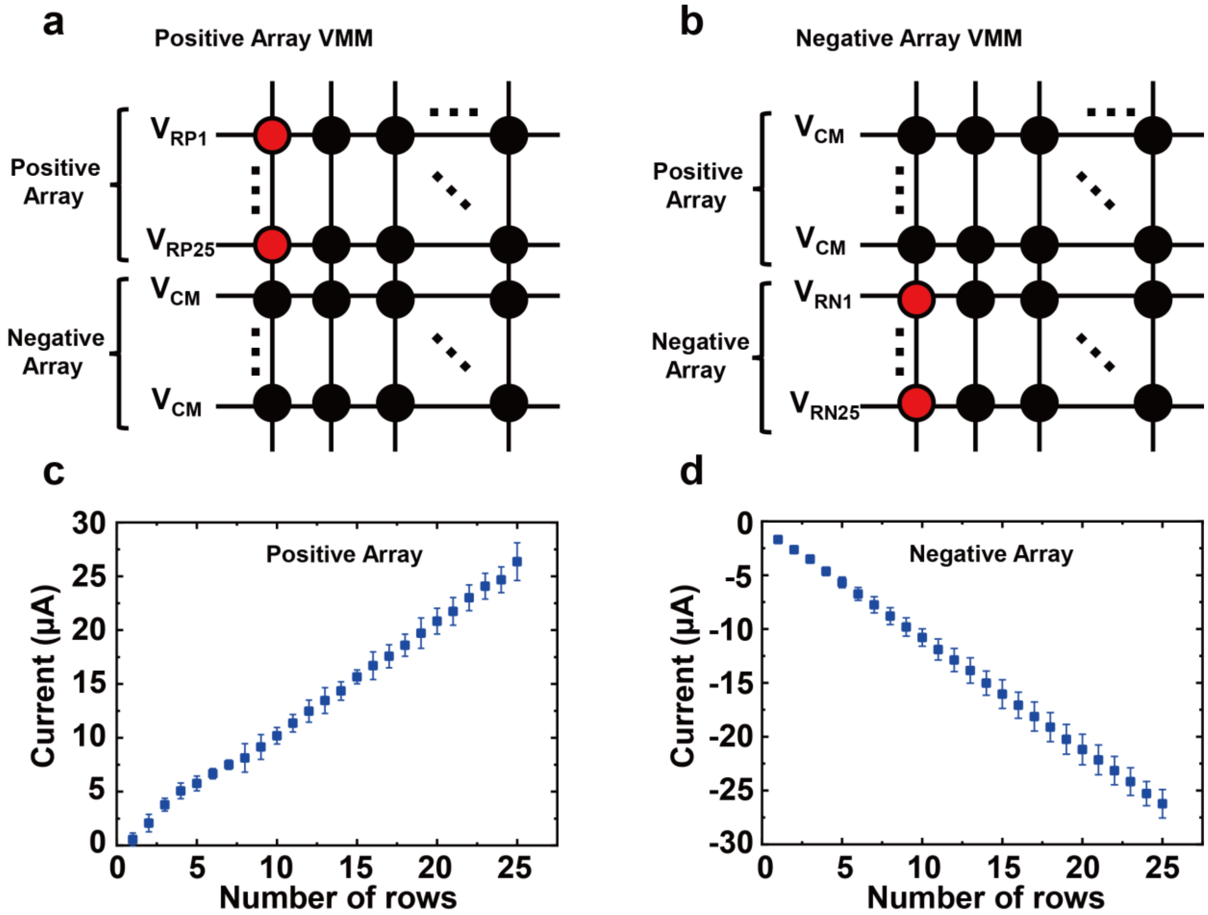

Figure S7. Measurement of vector-matrix multiplication based on the input vectors. a,b) Schematic diagrams of the measurement setup. In the measurement of the positive array, only V_{RP} is applied to the positive array, while V_{CM} is applied to the negative arrays. In the case of the negative array, only V_{RN} is applied. The input pattern increases cumulatively from 1 to 25, in which the numbers represent the number of rows connected to V_{RP} or V_{RN}. The conductance state of all memristors is set as the HRS, and the positive and negative arrays are measured separately. c,d) Measurement results of the positive and negative arrays. The *x*-axis represents the number of rows connected to V_{RP} or V_{RN}, and the *y*-axis represents the summed current. The measurement condition is that all memristors are in the HRS, in which conductance is distributed at approximately 2 μS. The results show the linear and uniform VMM performance, which depends on the gradually increasing input patterns.

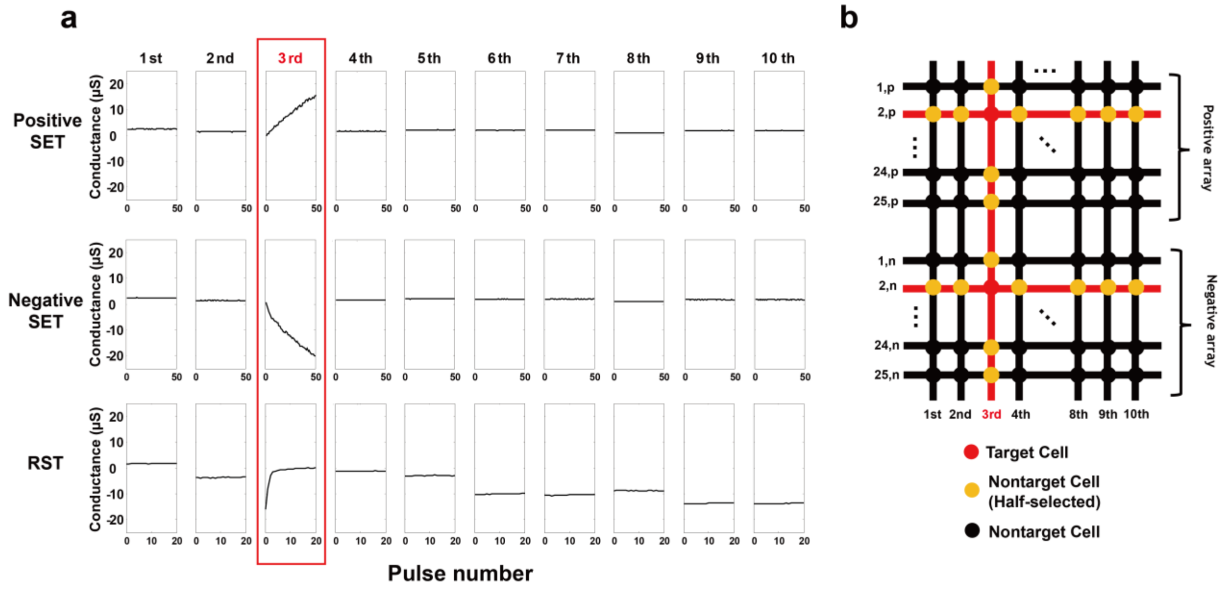

Figure S8. Monitoring of conductance switching during the writing process (positive SET, negative SET, RST) of the target memristor device. a,b) The conductance switching of the target devices located on the second row and third column in the positive and negative arrays during positive SET, negative SET, and RST. The *x*-axis and *y*-axis represent the number of writing pulses and the device conductance, respectively. When the status of unselected cells in applied row was measured in half-voltage scheme, the relevant device cells (10 numbers) just were kept in the initial OFF state as long as bias was not applied regardless of the status of the target cell. When the pulse is applied in the second row and third column, there are no analog transitions even in nearest neighborhood cells '2nd' and '4th' column. The suppressing of sneak path transport is associated with the threshold uniformity of the TiO_x memristor device, which entails the uniform SET response for all device cells in the array, where the range of switching voltage of the selected cell is not overlapped in half-voltage of nearest cells (Figure 1e). Also, the nonlinear pulse response of the device provides the dramatic reduction of switching current at low potentiation voltage (Figure 1g). Although the half-voltage effect is inevitable for neighboring cells in passive array structure, the transport along unselected lines can be effectively reduced and limited to nonlinear pulse response of each individual cell, resulting in excellent applicability of the TiO_x memristor device to the passive matrix architecture.

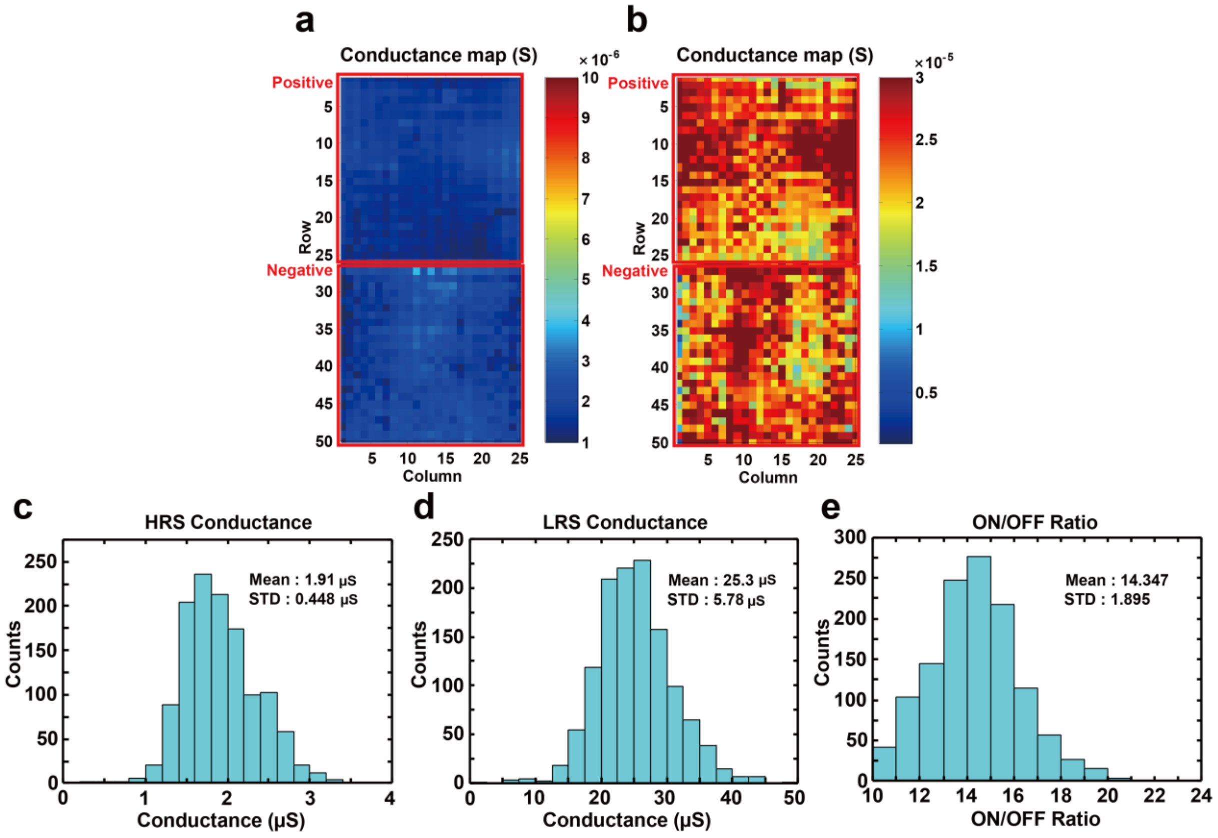

Figure S9. Statistical characteristics of the pair of 25 × 25 arrays. a,b) The conductance maps of the 50 × 25 array for all devices are in the HRS and LRS, respectively (1st ~ 25th row: positive array, 26th ~ 50th row: negative array). c) The HRS conductance distribution of 1000 devices. Their mean value and standard deviation are 1.91 μS and 448 nS, respectively. d) Histogram of the LRS conductance distribution. The mean value and standard deviation are 25.3 μS and 5.78 μS, respectively. e) Histogram of the ON/OFF ratio distribution based on the HRS and LRS conductance, in which the mean value and standard deviation are 14.347 and 1.895, respectively.

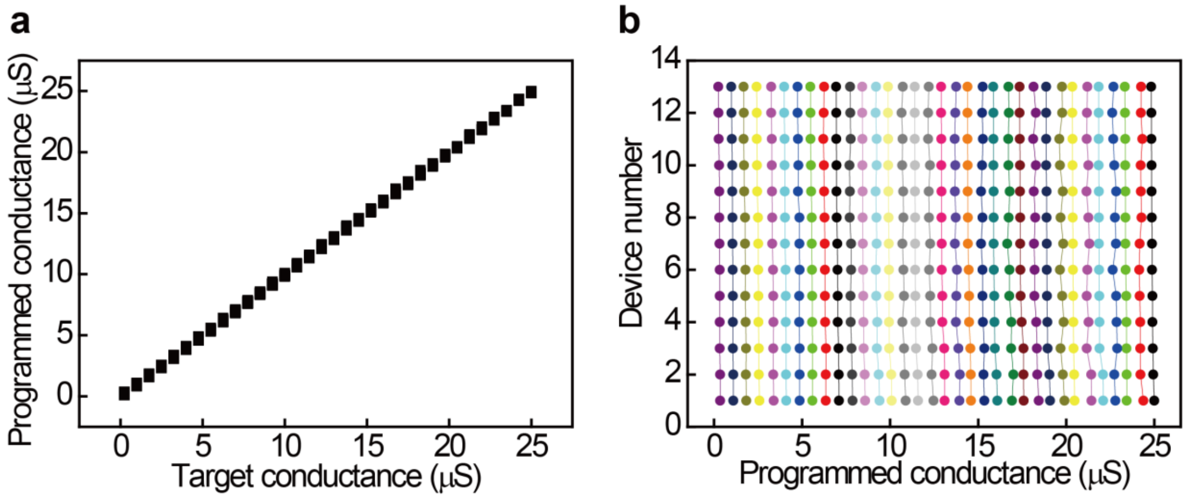

Figure S10. Experimental results for programming multi-level conductance state. a) 32-level programmed conductance states versus target conductance and b) their histogram for 13 randomly selected samples. The discrete 32 levels of conductance states, which are linearly spaced from 2 μS to 25 μS ensure that the device has at least 32-level of conductance resolution (equating 5-bit in the digital domain).

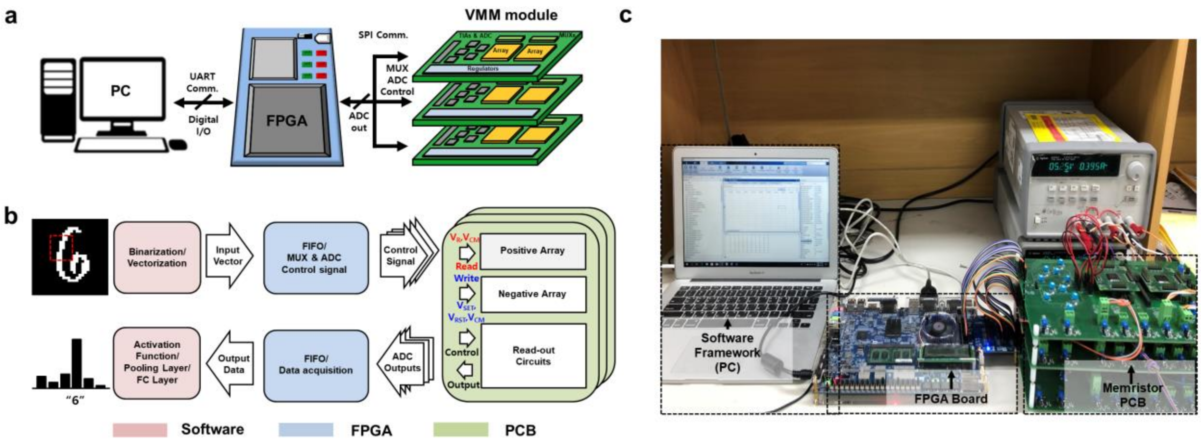

Figure S11. Experimental setup and operation details. a) Block diagram of the experimental environment, composed of a PC, an FPGA, and a VMM module with multiple PCBs. Input and output data are communicated via UART (universal asynchronous receiver/transmitter) serial communication between the PC and the FPGA board and SPI (serial peripheral interface) communication systems between the FPGA board and the PCBs. b) Flow diagram of the implemented CNN. In the PC, some parts of the CNN are implemented in the software, which also generates the input vector of the image sample or the number of writing pulses. The FPGA board generates a control signal for the electronic components and receives the

data from the PCBs. In the PCBs, iterative analog VMM computations and weight updates are performed. c) Photos of the experimental setup, including the PC, FPGA, and three PCBs with a memristor array.

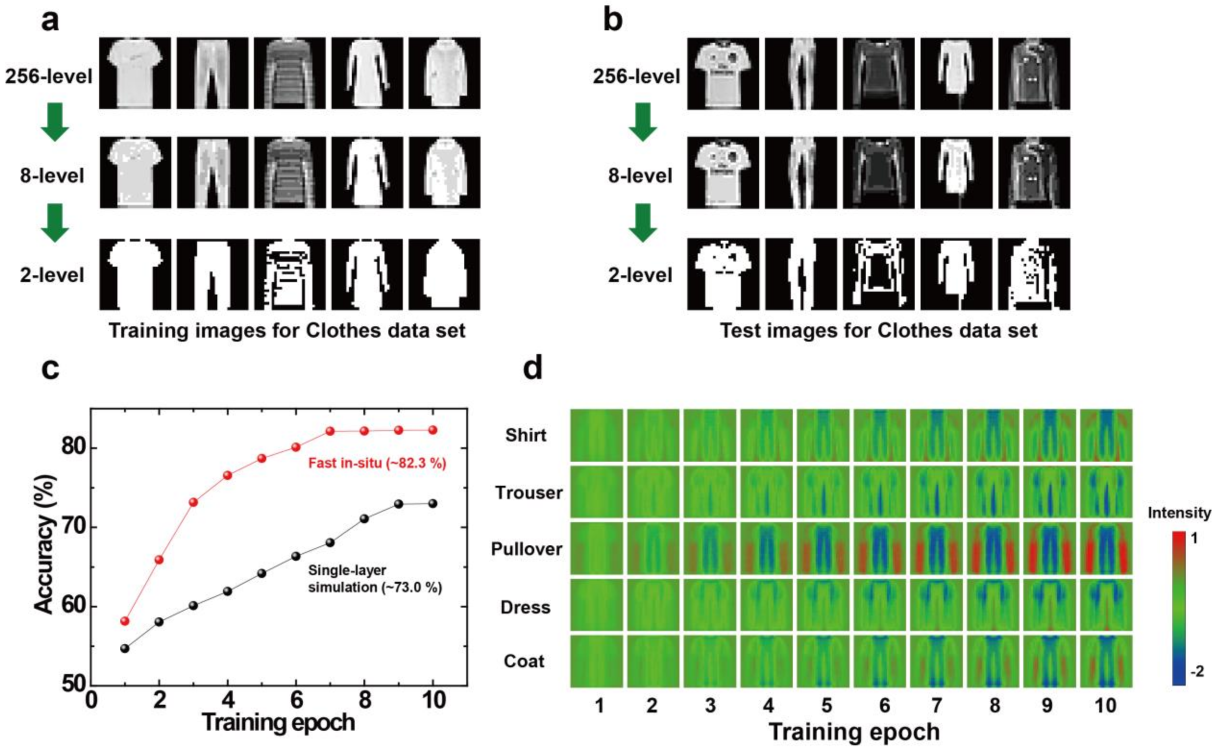

Figure S12. Clothes data recognition test. The quantization process of the original a) training images (30,000) and b) test images (5,000) in the Clothes data set as binary images. The original image data set are 28 pixels by 28 pixels with an 8-bit resolution. They were quantized as binary images based on the median pixel value while maintaining their image size in the digital computing platform. c) Classification accuracy for the Clothes data set using a single-layer simulation (black line, ~73.0 %) and FCIS training in the CNN hardware (red line, ~82.3 %). d) Reshaped 28 × 28 contour images of the synaptic weight propagation for the single-layer simulation at each training epoch, where the red and blue parts indicate the enhanced and decreased intensities for the synaptic weight, respectively.

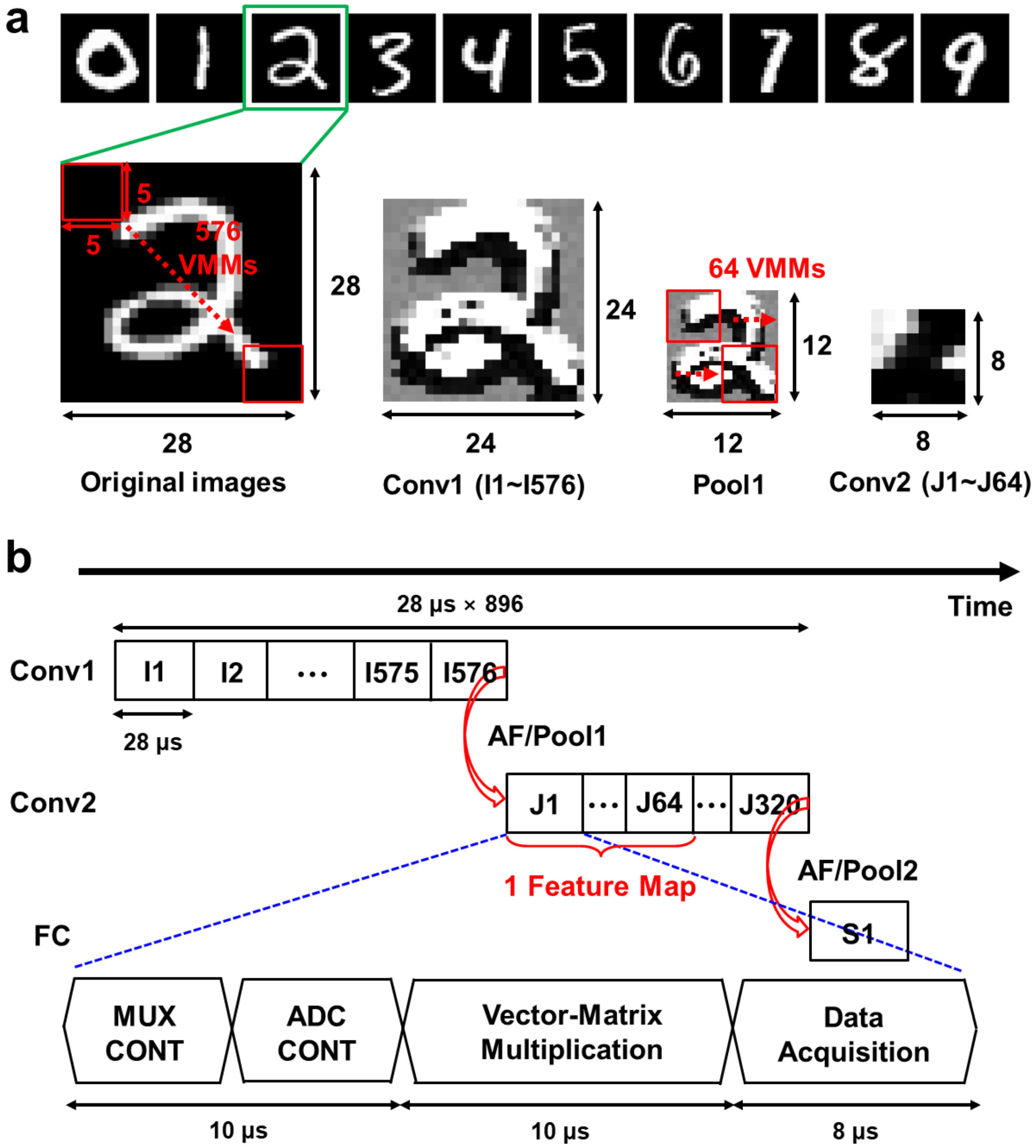

Figure S13-1. Inference process of the CNN. a) The change in the image data over multiple layers and the required number of VMM computations during the inference phase. The original MNIST image is 28 × 28 pixels, and 576 VMMs for the input vector *I* (*I1~I576*) are required in the first convolution layer with 5 × 5 kernel filters. The output map of the first convolution layer with the activation function (AF) is 24 × 24 pixels, which is resized as 12 × 12 pixels through the first pooling layer with 2 × 2 pooling filters. After 5 applications of 64 VMMs (for *J1~J320*) from the output feature map, the data size is reduced again as 8 × 8 and 4 × 4 through the second convolution layer and pooling layer, respectively. b) Illustration of the time span of an inference per image sample. The analog VMM for a set of input vectors

takes a total of 28 μs, including the transmission and reception of the serial control signal for MUXs and the ADC. Since 576 and 320 VMM computations are required in the first and second layers, the total time requirement for the inference of a sample image is 17,920 μs, which is sufficiently lower than relaxation time scale for each analog conductance state (Figure 1i) to perform the reliable in-situ training process.

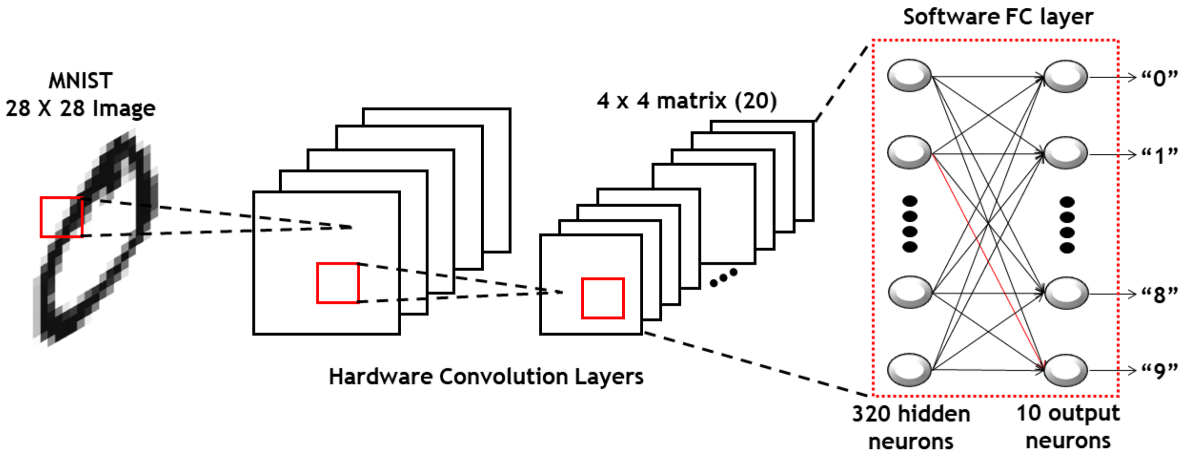

Figure S13-2. Software implemented fully connected (FC) layer. The 20 number of 4 × 4 output feature maps from the 2nd pooling layer are flatted as a vector form with 320-length and applied to the FC layer. The size of the weight matrix of the FC layer is 320 × 10 and the weight value is trained in software. After the VMM between the input data and weight matrix followed by the activation function transformation, output vector with 10-length is produced, and then, the final recognition answer is determined according to the index of neuron with the maximum value among the 10 output values.

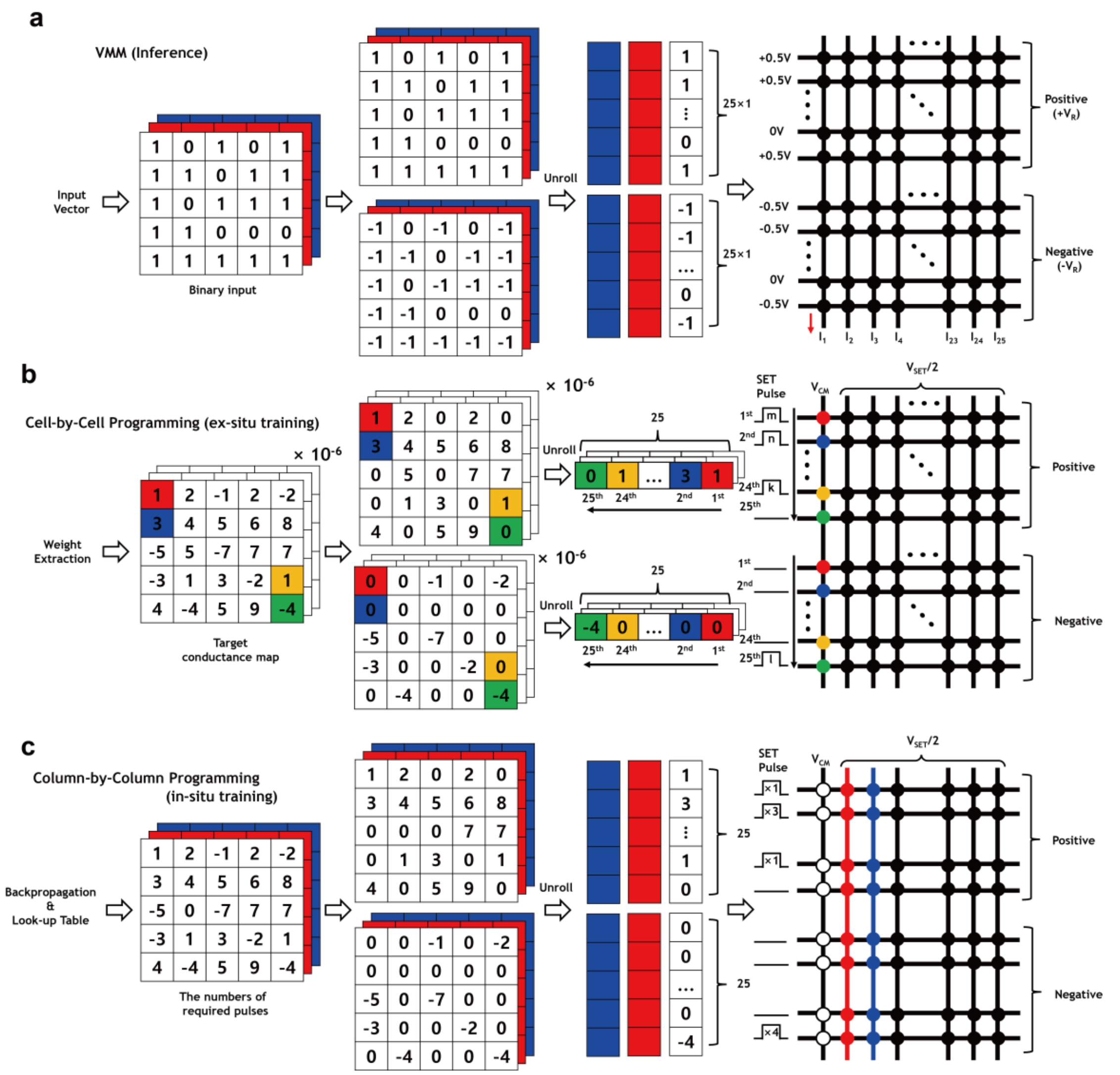

Figure S14. Three different input data flows depending on the operations of the CNN. a) The data flows during the inference phase. The 5 × 5 partial binary image matrix is divided into positive and negative matrices, which are converted to 25-length vectors. The input vectors are applied to the memristor arrays as either V_{RP} or V_{RN}, which are 0.5 V and -0.5 V relative to V_{CM} (2.5 V), respectively. The current is summed using equation (1) in the main text. b) The data flow during the ex-situ training. After ex-situ training in an external computer, the weight map is extracted in units of the conductance of the memristor. The extracted conductance map is divided into positive and negative matrices, and elements with opposite polarity are replaced with 0 in each matrix. Here, the zeros means that there is no write process in the cell at that location. The write process precedes sequentially in each device. c) The data flow during the in-situ training. After inference, the numbers of writing pulses for the weight update of each memristor device are determined through the backpropagation algorithm and look-up table (LUT). The matrix of the number of writing pulses is also divided into positive

and negative matrices, where negative polarity means that the number of pulses is applied to the device on the negative array. The matrices are converted to vectors, and the weight updates are achieved in row-parallel manner by applying writing pulses in parallel to each row.

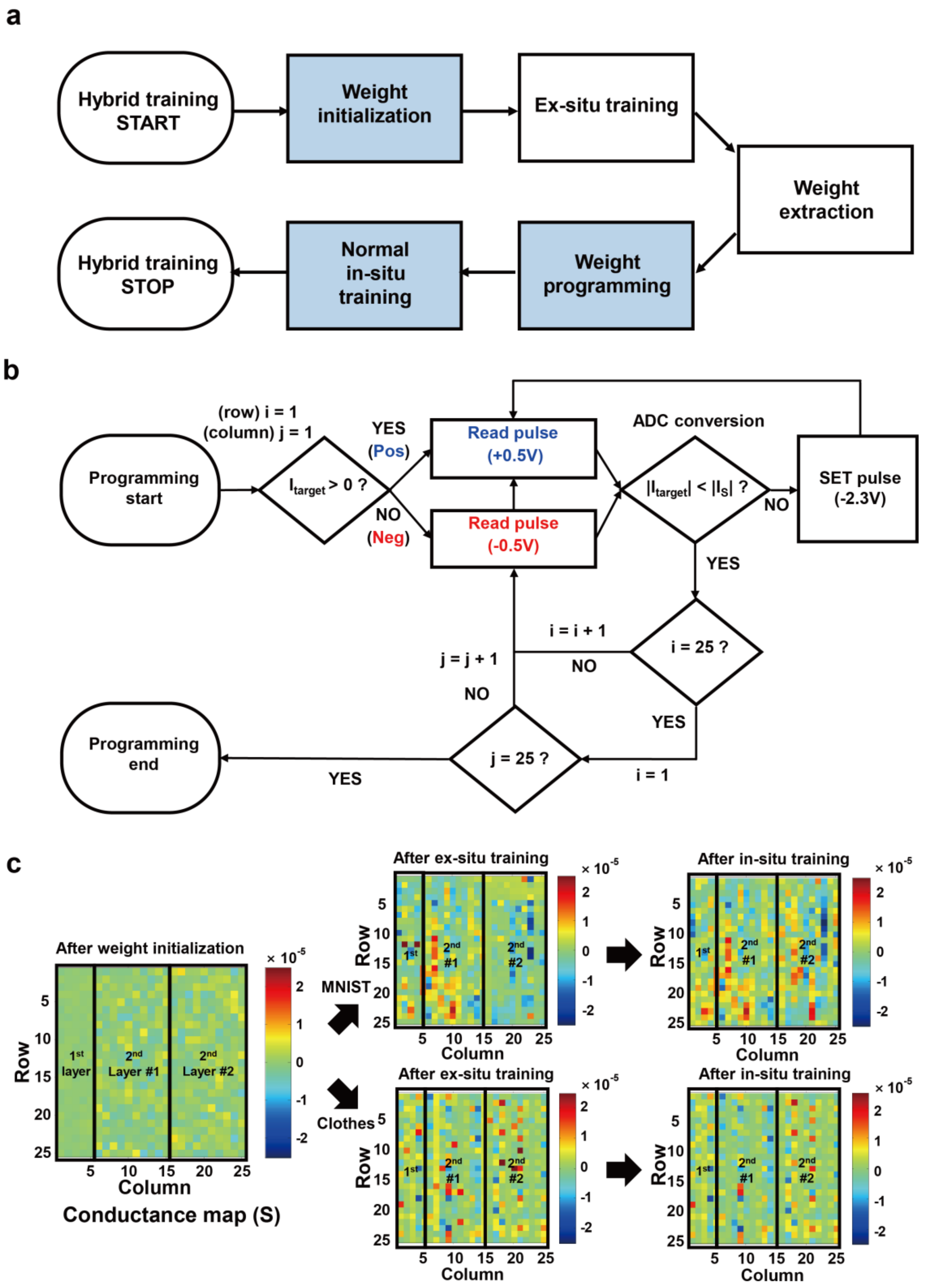

Figure S15. Illustration of hybrid training and encoding weight to the conductance. a) Flow diagram of hybrid training. The highlighted box represents the hardware-implemented process. b) Flow diagram of the process to program the conductance of the 25 × 25 memristor array to the target conductance map, which is based on the 1W-1R programming scheme. c) Transition of the conductance map in each step of hybrid training. During the weight initialization process, the conductance of all memristors is reset to the HRS in the weight initialization process. After weight programming through ex-situ training, the conductance is finely adjusted for further optimization of the classification accuracy by in-situ training.

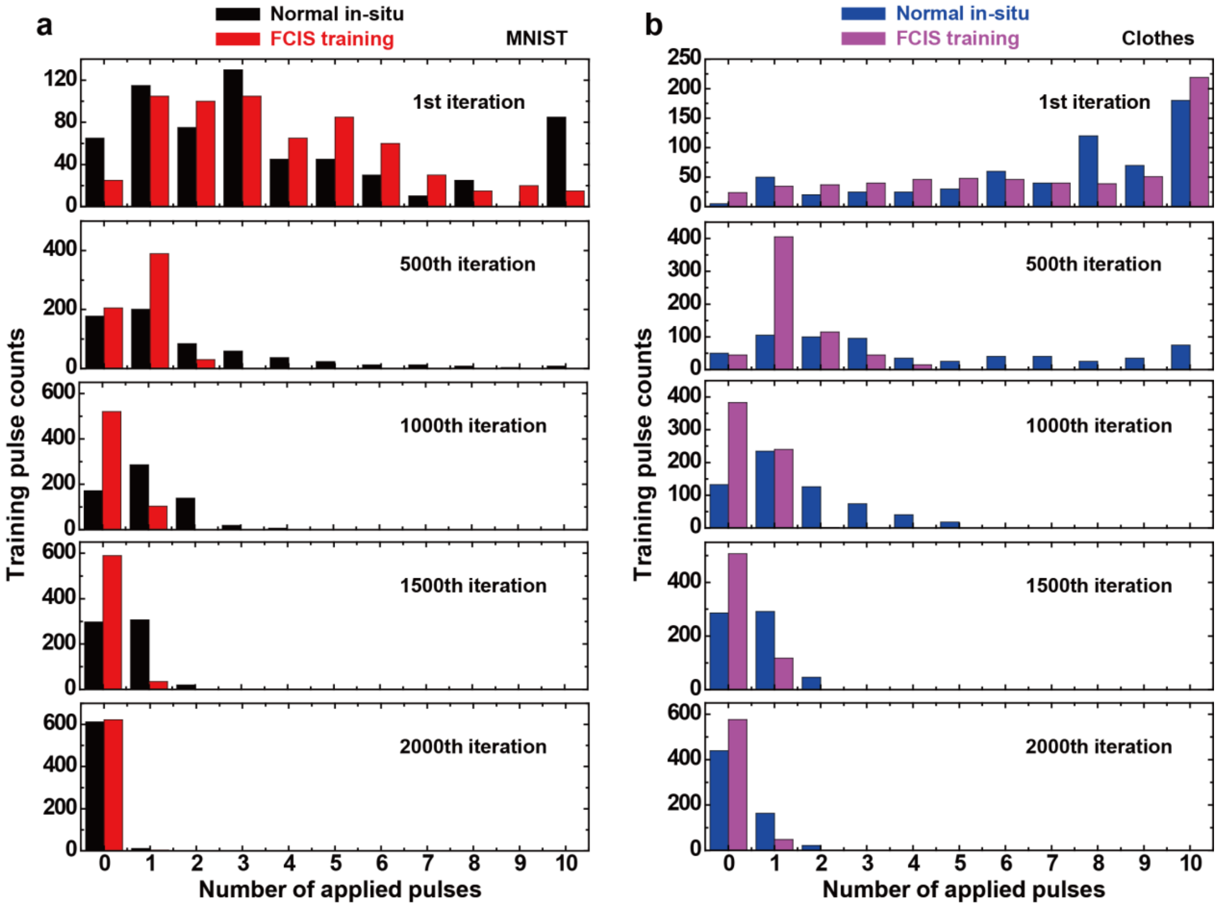

Figure S16. Histogram plot of the number of applied writing pulses at the 1st, 500th, 1000th, 1500th, and 2000th iterations. Distribution of the number of pulses a) when trained on MNIST images and b) when trained on Clothes images. The decrease in the number of pulses with increasing number of iterations shows the convergence of the loss function mitigating conductance error for each cell and training effectiveness of pulse number modulation. The histogram indicates that FCIS training using a high rate of conductance change at the initial

iteration has high training efficiency with a relatively fewer number of pulses than required in normal in-situ training. The number of writing pulses is converted from the amount of weight update, ΔW, determined in equation (5) in the main text through the LUT (Table S1).

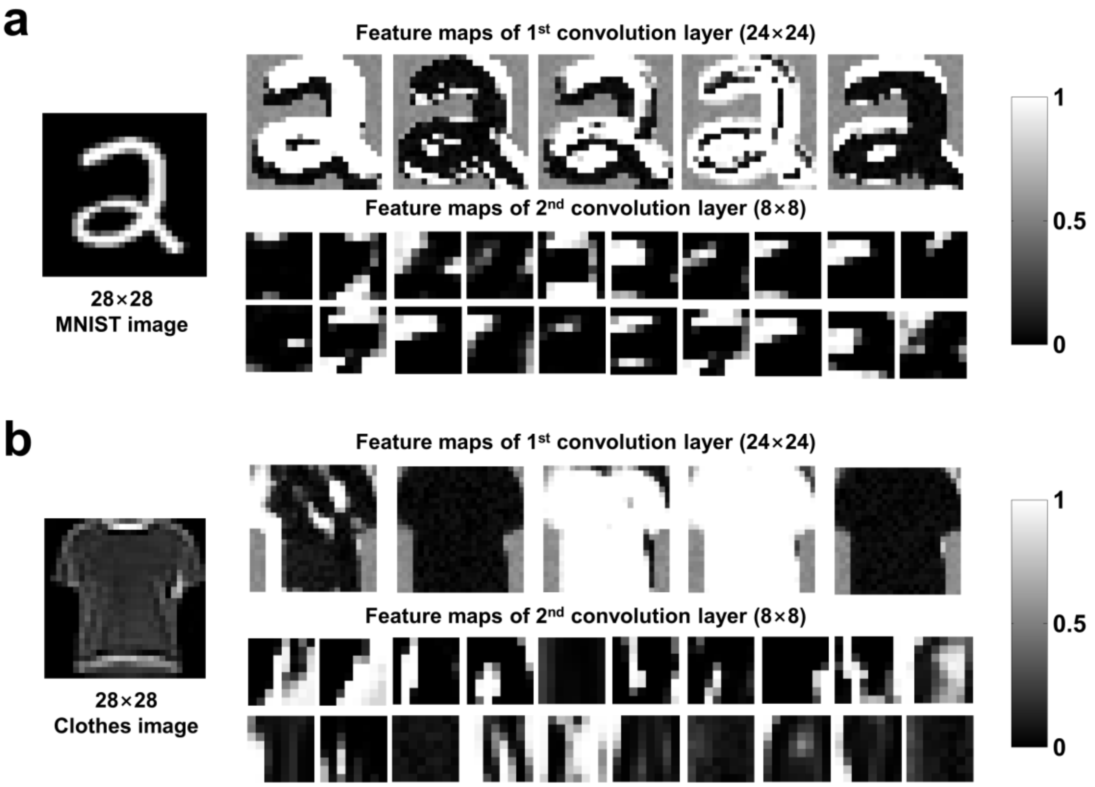

Figure S17. Intermediate sigmoid output maps of the 1st and 2nd convolution layers. Example of sigmoid output maps for the 1st and 2nd convolution layers a) for the MNIST image “2” and b) for the Clothes image “shirt”. Each pixel of data is received as a 12-bit digital signal and reconstructed as 24 × 24 and 8 × 8 images by the experimental PC. Since there are 5 kernel matrices and 20 kernel matrices in the 1st and 2nd convolution layers, respectively, 5 and 20 different feature output maps are produced.

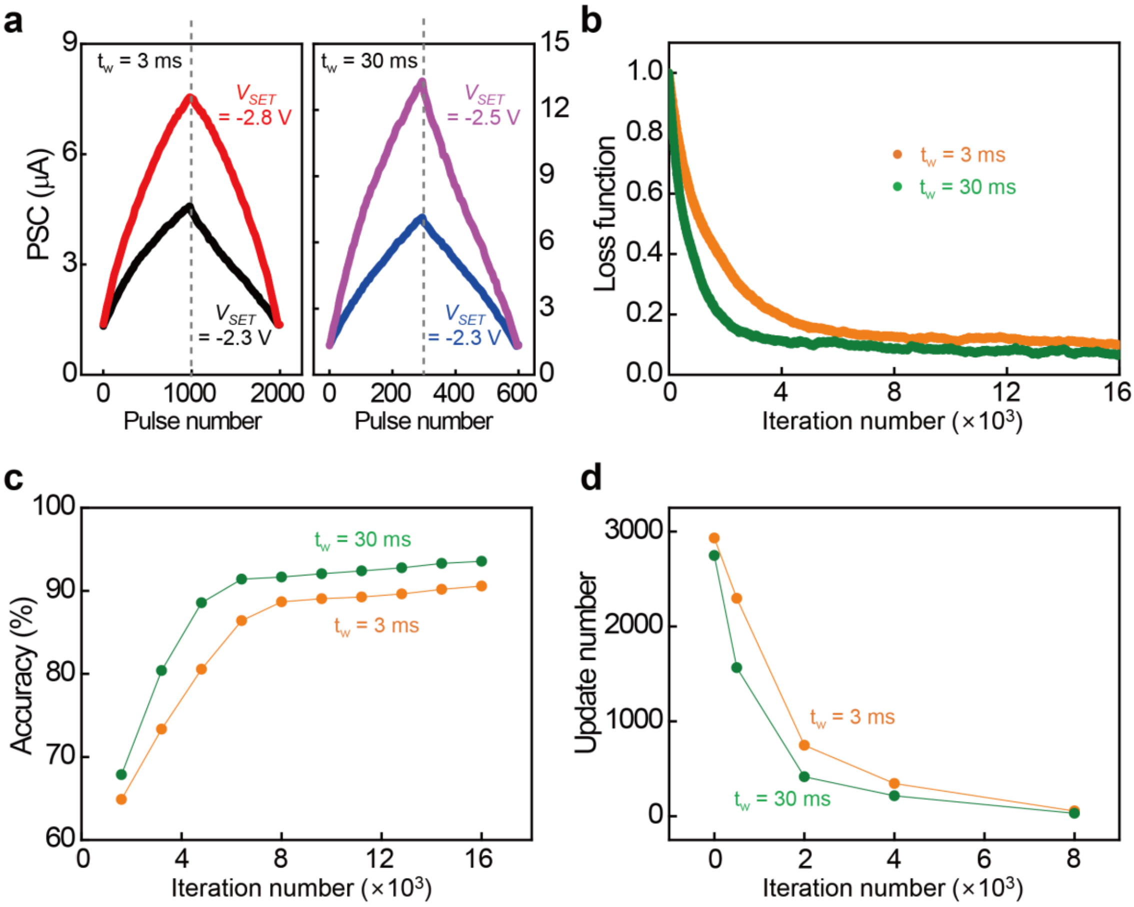

Figure S18. Measurement and training results with short pulse width (3 and 30 ms). a) Effective LTP/LTD functions triggered by positive and negative devices with -2.8 V and -2.3 V SET pulse with 3 ms pulse width (left), and with -2.5 V and -2.3 V SET pulse with 30 ms pulse width (right). b) The normalized loss function of the CNN for FCIS training with 3 ms (yellow line) and 30 ms (green line) pulse width. The loss function with 30 ms pulse width reaches the saturation point of the training after around 4,000, which is half of the required number of iterations to the saturation point with 3 ms pulse width. c) The change of classification accuracy over 16,000 iterations, where the classification accuracy is evaluated after every 400 iterations and the final classification accuracy is 93.5 % and 90.5 % with 30 ms and 3 ms of pulse width, respectively. d) The total number of required training pulses at the initial, 500th, 2,000th, 4,000th, and 8,000th iteration. In our study, the switching constraint of the memristor array device was determined to exhibit most appropriate analog switching properties (for example: dynamic range, asymmetry, and repetitive endurance) for high performance of hardware in-situ training.

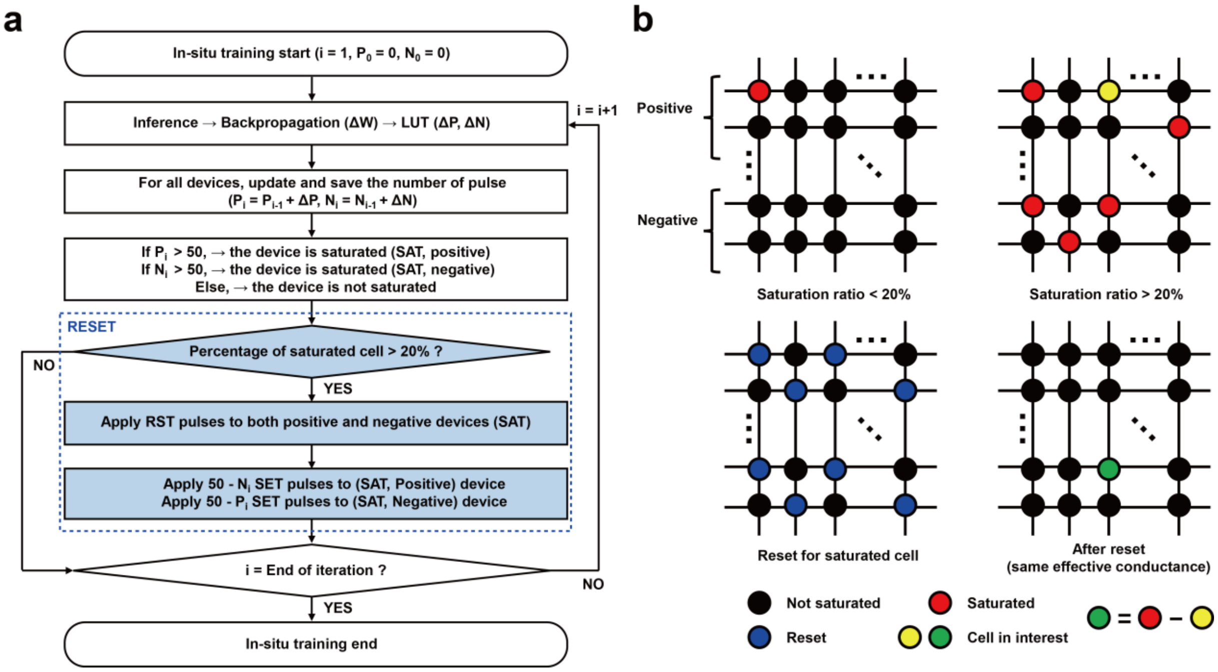

Figure S19. Conductance RST process during in-situ training of CNN hardware. a) A flow diagram of the RST process highlighted in blue box during in-situ training. b) The description for the change in the conductance state following the RST process. The RST process is performed on the positive and negative devices one of which is saturated with LRS after the LUT conversion. Whether the device is saturated with LRS depends on whether the number of pulses accumulated until the current iteration, P_i (positive pulse) and N_i (negative pulse), exceeds 50. If the percentage of the saturated device is over the 20, the conductance of both saturated device and counterpart device are reset and reprogrammed to the previous effective conductance value by applying either (50- N_i) positive pulses or (50- P_i) negative pulses.

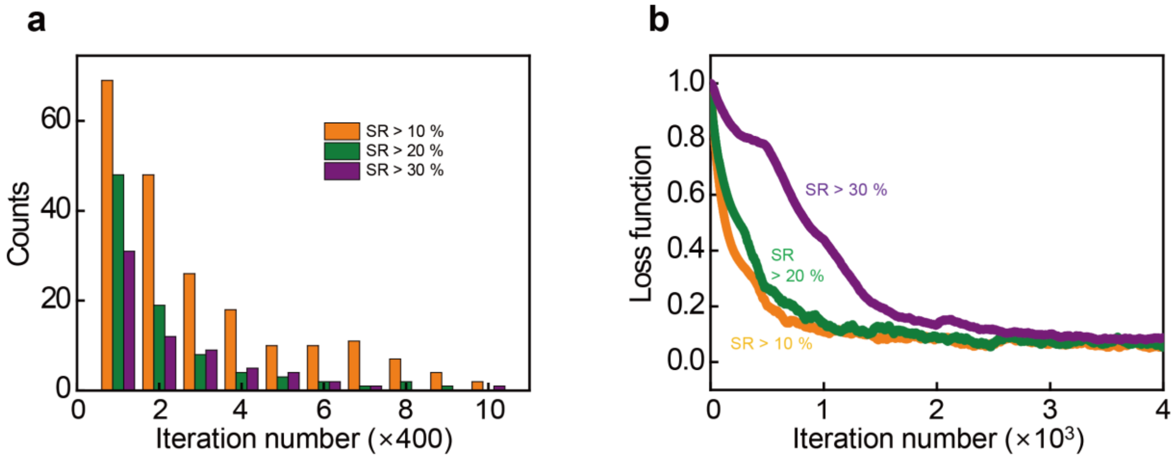

Figure S20. Experimental comparison of training performance for RST depending on the saturation ratio (SR). a) The required number of RST process for entire training iterations and b) transition of normalized loss function of the CNN during the in-situ training according to the SR. The result of RST for 20 % of SR shows similar decrease in loss function comparing the result of RST for 10 % rate, despite requiring around 52 % less the number of the RST process. If either positive or negative device is saturated to LRS during training, the effective conductance moves to the only one direction, increasing or decreasing. Since those asymmetric weight updates in some saturated devices can degrade the training performance, the conductance RST process should be accompanied to complete the training. Note that since there is no conductance read process during in-situ training, whether the device is saturated or not is determined by monitoring the accumulated number of pulses until the current iteration instead of reading the amount of the conductance change after each iteration. Depending on the SR, which is the percentage of the saturated device in the overall array, the RST process is performed automatically between training iterations by applying 5 RST pulses to the each saturated device in a row-parallel manners and the RST devices are re-programmed to the previous effective conductance value. The optimal SR for RST is determined as 20 % according to the trade-off between the total number of RST process performed until the final iterations and the required number of iterations to reach the converged loss function.

a

	8-bit Digital	Memristor (1μS)
Multiplication/ Addition (VMM)	0.2pJ / 0.03pJ	10fJ
Memory Access (Programming)	20pJ/bit, 160pJ	4nJ (1ms), 40nJ (10ms)

Epoch	8-bit Digital		Memristor			
	VMM (μJ)	PROG (mJ)	VMM (μJ)	PROG (10ms, mJ)	PROG (1ms, mJ)	PROG (1ms, FCIS, mJ)
1	0.272	0.12	0.0767	0.443	0.165	0.0460
2	0.543	0.24	0.042	0.735	0.259	0.164
3	0.815	0.36	0.0639	1.119	0.302	0.28
4	1.09	0.48	0.0834	1.473	0.273	0.365
5	1.36	0.6	0.103	2.162	0.554	0.411
6	1.63	0.72	0.153	2.497	0.718	0.443
7	1.90	0.84	0.175	2.892	0.908	0.455
8	2.17	0.96	0.192	3.35	1.071	0.477
9	2.44	1.08	0.210	3.83	1.113	0.486
10	2.72	1.2	0.232	4.425	1.392	0.490

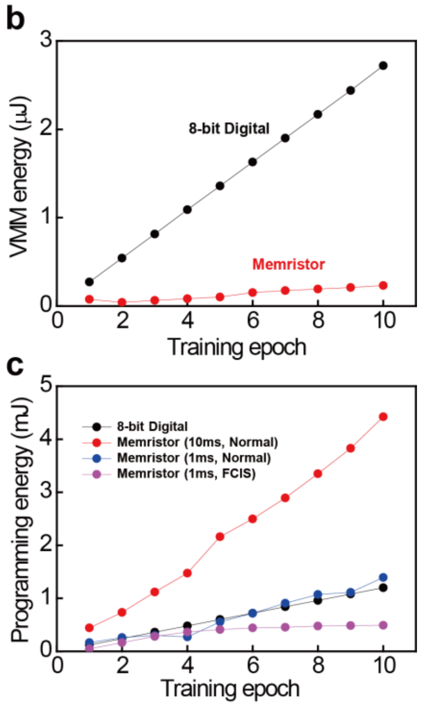

Figure S21. Comparison for the training energy of 8-bit digital memory and analog memristor. a) Simulation results of total energy consumption over training epochs for b) VMM computation and c) memory access (programming, PROG) per unit device.

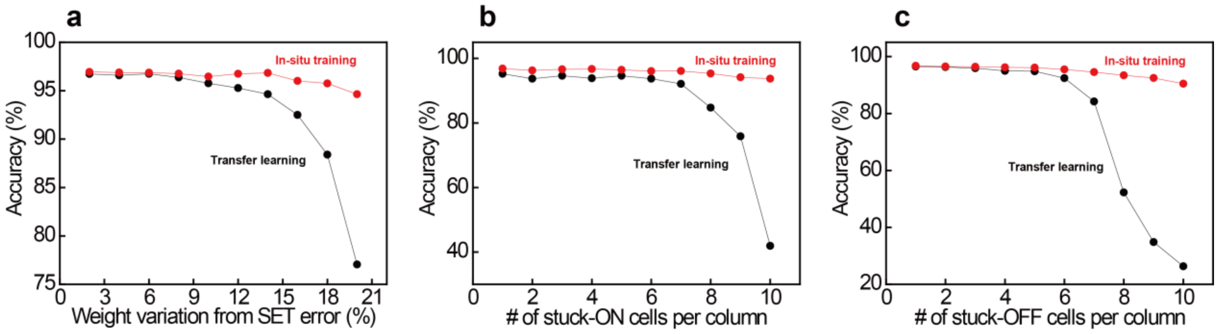

Figure S22. Classification accuracy of in-situ training and transfer learning with device imperfections as a) weight variation, b) number of stuck-ON cells, and c) number of stuck-OFF cells per column.

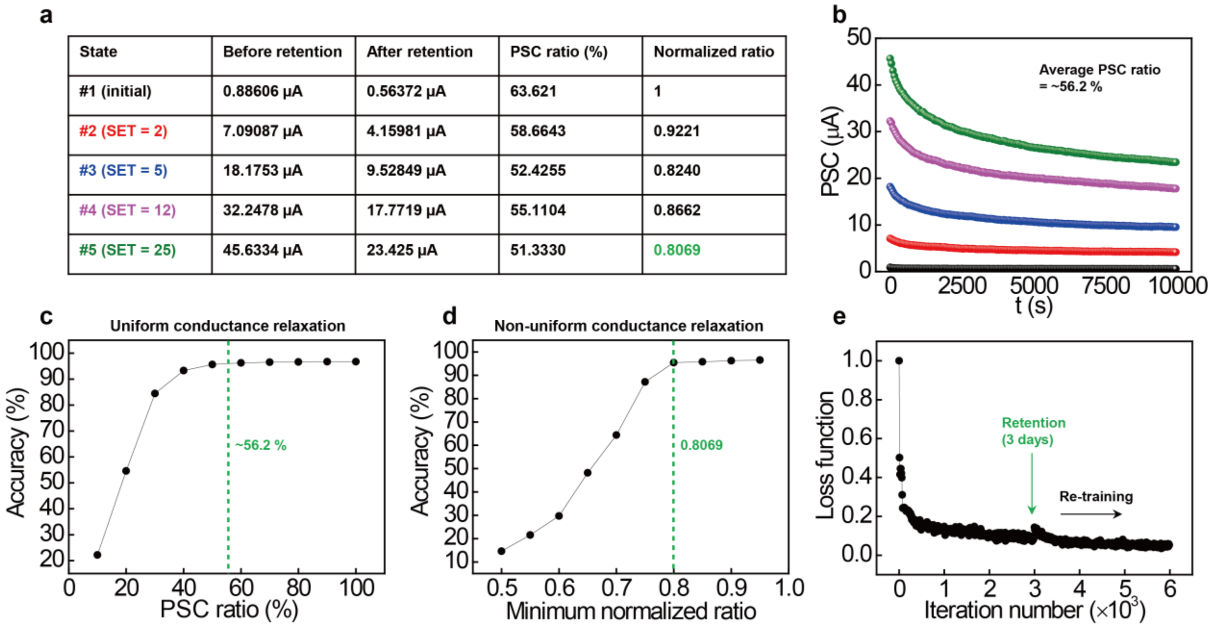

State	Before retention	After retention	PSC ratio (%)	Normalized ratio
#1 (initial)	0.88606 μA	0.56372 μA	63.621	1
#2 (SET = 2)	7.09087 μA	4.15981 μA	58.6643	0.9221
#3 (SET = 5)	18.1753 μA	9.52849 μA	52.4255	0.8240
#4 (SET = 12)	32.2478 μA	17.7719 μA	55.1104	0.8662
#5 (SET = 25)	45.6334 μA	23.425 μA	51.3330	0.8069

Figure S23. The effect of retention variation of the TiO_x memristor devices. a) PSC values and variation for different analog states in b) retention test. Classification accuracy for c) uniform and d) non-uniform conductance relaxation. e) The result of hardware in-situ training with 3 days of retention time interval at 3,000 of iteration number.

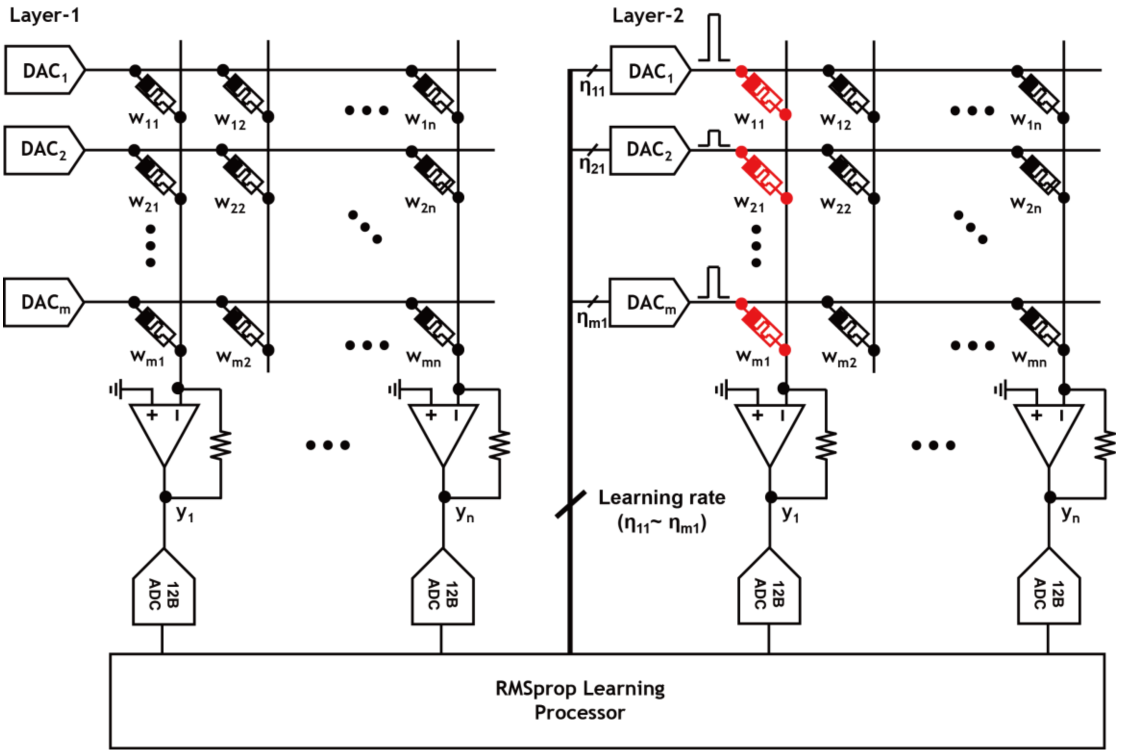

Figure S24. Schematic of RMSprop hardware.

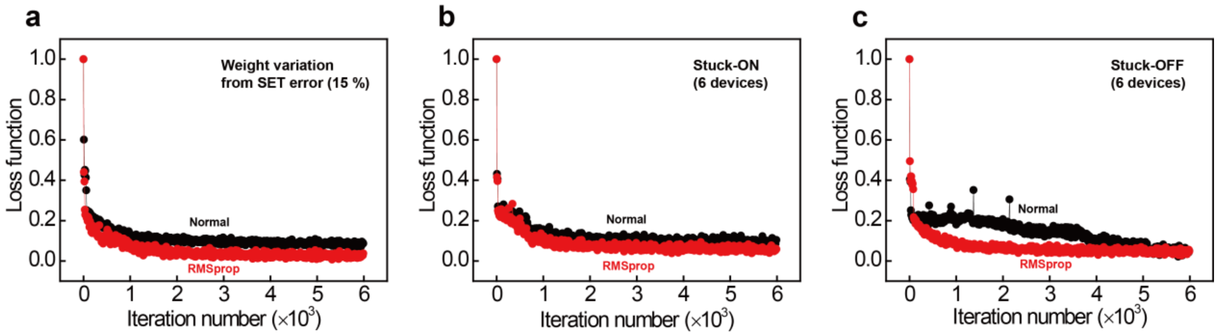

Figure S25. The comparison of training results between the normal training and RMSprop training for the a) weight variation, b) stuck-ON, and c) stuck-OFF.

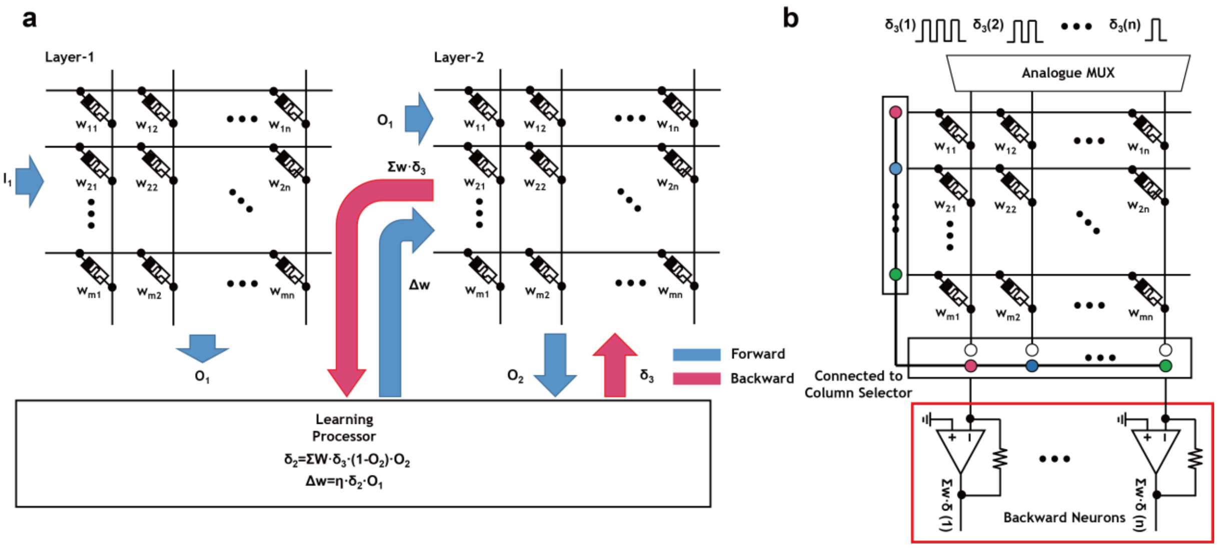

Figure S26-1. Backpropagation scheme of convolution module. a) Forward/backward VMM between two layers for weight update and b) backward VMM using a convolution module.

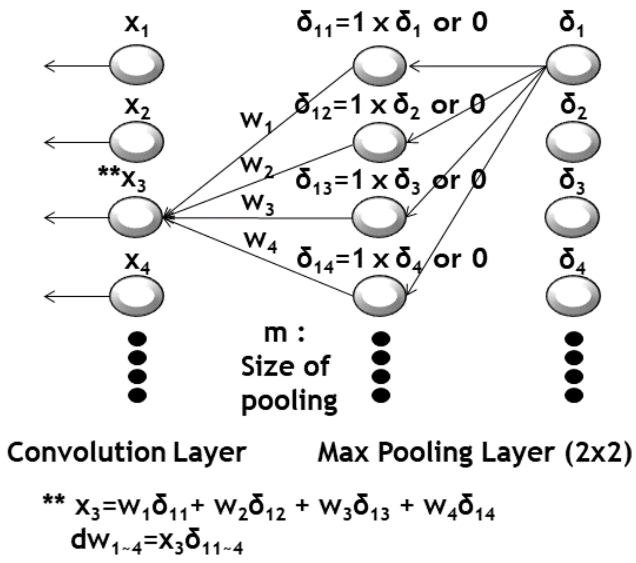

Figure S26-2. Backpropagation in convolution and pooling layer. The local gradient at the max pooling layer is 1 or 0, and when assumed that gradient x_3 is connected to the filter w_1, w_2, w_3, and w_4 at the convolution layer, the gradient x_3 is determined by sum of the gradient δ and local gradient w.

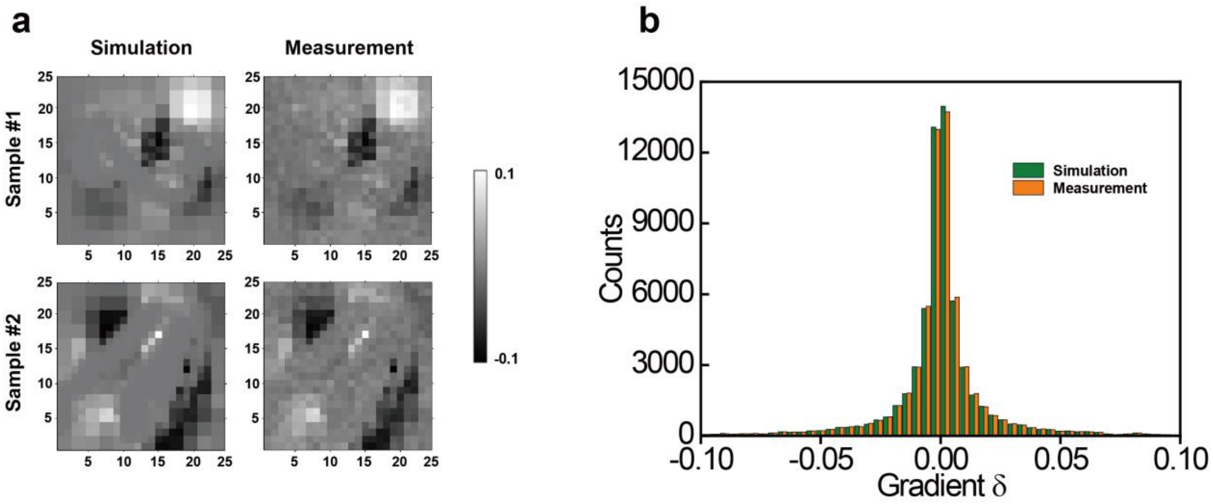

Figure S27. Simulation and measurement results of backpropagation. a) Comparison of backpropagated gradient vector (δ) of 2nd layer in simulation and measurement for same input pattern. b) Histogram of δ accumulated for 100 input samples in simulation and measurement.

Table S1. Look-up Table (LUT) for training the conductance of a memristor array. The LUT converts the amount of weight update ΔW (see (5) in Methods) to the number of training pulses. If $\Delta W > 0$, the relevant number of SET pulses are applied to the positive array, and if $\Delta W < 0$, it is applied to the negative array in the same manner. Considering the range of ΔW computed by the backpropagation algorithm, the maximum range of the LUT is set symmetrically to positive and negative from -2.5×10^{-7} to 2.5×10^{-7}. The maximum number of training pulses per iteration is optimized as 10 through trial and error. For the encoded weight of the memristor model in FC layer, the converted number of pulses is re-converted to ΔW according to the intervals.

**Range of $\lvert\Delta W\rvert$**	**# of pulses**
$\lvert\Delta W\rvert \times 10^9 < 10$	**0**
$10 \le \lvert\Delta W\rvert \times 10^9 < 20$	**1**
$20 \le \lvert\Delta W\rvert \times 10^9 < 30$	**2**
$30 \le \lvert\Delta W\rvert \times 10^9 < 40$	**3**
$40 \le \lvert\Delta W\rvert \times 10^9 < 50$	**4**
$50 \le \lvert\Delta W\rvert \times 10^9 < 70$	**5**
$70 \le \lvert\Delta W\rvert \times 10^9 < 100$	**6**
$100 \le \lvert\Delta W\rvert \times 10^9 < 140$	**7**
$140 \le \lvert\Delta W\rvert \times 10^9 < 190$	**8**
$190 \le \lvert\Delta W\rvert \times 10^9 < 250$	**9**
$250 \le \lvert\Delta W\rvert \times 10^9$	**10**

Table S2. Table for comparing training efficiency depending on the pulse width of the training pulse and the presence of the learning rate adjustment. Considering the increased number of pulses to HRS form LRS, total 16,000 iterations are performed in the training with 30 and 3 ms of pulse width, and in the results, more than 4 and 6 times of the total number of training pulse compared to the training with 500 ms pulse width are required, respectively. Given the same number of training pulses and iteration required for training, the total energy consumption for training is expected to be proportional to the pulse width of the training pulse. However, in terms of power efficiency, since only the number of iteration and training pulse affect the performance with the fixed pulse amplitude, the power efficiency with 500 ms pulse width is 12 times higher than that with 3 ms pulse width.

Type	V_{SET} (V)	V_{RST} (V)	Pulse width (ms)	Iteration number	Update number
Normal in-situ training	-2.3	2.5	500	4,000	1,664,581
FCIS training	-2.3 / -2.5	2.5	500	4,000	875,403
FCIS training	-2.3 / -2.5	2.5	30	16,000	3,684,148
FCIS training	-2.3 / -2.8	2.5	3	16,000	5,378,317

Table S3-1. The research progress of TiO_x-based RRAM and memristor devices.

	Type	Structure	Threshold deviation	Device yield	Repetitive stability	*x*-value (in TiO_x)	Forming	Elapsed time	Note
**Our work**	**Memristor**	**Crossbar array**	**~2.7 %**	**> 99 % (120/120)**	**~3000 spikes**	**~2.03**	**Not needed**	**6 month**	**25 × 25 array**
**D.-H. Kwon *et al.* Atomic structure of conducting nanofilaments in TiO2 resistive switching memory. *Nat. Nanotechol.* 5, 148–153 (2010).**	**RRAM**	**Single cell junction**	**-**	**-**	**-**	**~2.1**	**Essential**	**-**	**Conducting filament structure**
**S.-J. Park *et al.* In situ control of oxygen vacancies in TiO2 by atomic layer deposition for resistive switching devices. *Nanotechnology* 24, 295202 (2013).**	**RRAM**	**Single cell junction**	**> 25%**	**-**	**-**	**1.62~1.70**	**Essential**	**-**	**Control of *x*-value**
**D. B. Strukov *et al.* The missing memristor found. *Nature* 453, 80-83 (2008).**	**Memristor**	**Single cell junction**	**-**	**-**	**-**	**-**	**-**	**-**	**Emulating the synaptic function**
**K. Szot *et al.* TiO2—a prototypical memristive material. *Nanotechnology* 22, 254001 (2011).**	**Memristor**	**Single cell junction**	**-**	**-**	**-**	**1.5~2.0**	**Essential**	**-**	**Topical review**
**J. J. Yang *et al.* Dopant Control by Atomic Layer Deposition in Oxide Films for Memristive Switches. *Chem. Mater.* 23, 123–125 (2011).**	**Memristor**	**Crossbar array**	**-**	**-**	**-**	**~2.0**	**Essential**	**-**	**Effect of x-value**
**K. Seo *et al.* Analog memory and spike-timing-dependent plasticity characteristics of a nanoscale titanium oxide bilayer resistive switching device. *Nanotechnology* 22, 254023 (2011).**	**Memristor**	**Single cell junction**	**-**	**-**	**-**	**1.77~1.83**	**-**	**-**	**Emulating the synaptic function**
**J. Park *et al.* Nitrogen-Induced Enhancement of Synaptic Weight Reliability in Titanium Oxide-Based Resistive Artificial Synapse and Demonstration of the Reliability Effect on the Neuromorphic System. *ACS Appl. Mater. Interfaces* 11, 32178–32185 (2019).**	**Memristor**	**Crossbar array**	**~6.21 %**	**-**	**~5000 spikes**	**~1.8**	**Essential**	**-**	**10 devices measurement**
**S. Kim *et al.* Flexible Memristive Memory Array on Plastic Substrates. *Nano Lett.* 11, 5438–5442 (2011).**	**Memristor**	**1T-1M**	**-**	**~60 % (35/64)**	**~100 cycles**	**-**	**-**	**-**	**8 × 8 NOR type array**
**H. Abbas *et al.* A memristor crossbar array of titanium oxide for non-volatile memory and neuromorphic applications. *Semicond. Sci. Technol.* 32, 065014 (2017).**	**Memristor**	**Crossbar array**	**-**	**-**	**~30 cycles**	**-**	**-**	**-**	**3 × 3 array**

Table S3-2. The research progress of representative metal oxide-based memristor devices.

	Switching material	Structure	Threshold deviation	$\|V_{TH}\|$ (V) (pulse/DC)	Device yield	Repetitive stability	Forming	ON/OFF (analog)	Nonlinear pulse response	Note
Our work	TiO_x	Crossbar array	~2.7 %	1.55/ 2.35	> 99 % (120/120)	~3000 spikes	Not needed	~25.7	Achieved	25 × 25 array
X. Zhan et al. *Nanotechnology* 32, 35LT01 (2021).	AlO_x	Single cell junction	-	1.5~3/ 1.5~3	-	-	Not needed	~3	Achieved	ALD condition
X. Huang et al. *Appl. Phys. Lett.* 116, 173504 (2020).	AlO_x	Single cell junction	Broad (0.5~1.5 V)	-	-	-	Essential	~103	-	8-state
E. Covi et al. *IEEE International Symposium on Circuits and Systems (ISCAS)*, pp. 393-396, doi: 10.1109/ISCAS.2016.7527253 (2016).	HfO_2	Single cell junction	-	0.55/ 0.6	-	-	Essential	~2	-	SNN learning
S. Chandrasekaran et al. *Nanotechnology* 30, 445205 (2019).	HfO_2	Crossbar array	-	2.5/ 2~3	-	~450 spikes	Essential	~2.5	-	8×8 array
Z. Li et al. *IEEE Electron Device Lett.* 40, 1068 (2019).	HfO_x	Single cell junction	~3.3 %	0.4~0.9/ 0.6~1.5	-	~800 spikes	Essential	~5	-	4 device evaluation
W. Wang et al. *IEEE Electron Device Lett.* 40, 1407 (2019).	NbO_x	Crossbar array	-	5/ 6	-	-	-	~4.5	-	Single array
S. Deswal et al. *AIP Advances* 9, 095022 (2019).	NbO_x	Crossbar array	-	6~10/ 5~11	-	-	-	~10	-	5×5 array
K. Moon et al. *IEEE Electron Device Lett.* 37, 1067 (2016).	PCMO	Crossbar array	-	3/ 1.5~3.5	-	-	-	~10	-	5-bit MLC by SRDP
D. Lee et al. *Appl. Phys. Lett.* 106, 113701 (2015).	PCMO	Single cell junction	-	2.5/ 2~3	-	-	-	~23	-	Effect of electrode
K. M. Kim et al. *Sci. Rep.* 6, 20085 (2016).	TaO_x	Crossbar array	Broad (3.5~4.5 V)	3.5/ 3.5~4.5	-	-	-	10~50	-	Effect of voltage divider
A. Saleem et al. *Appl. Phys. Lett.* 118, 112103 (2021).	TiW/TaO_x	Single cell junction	-	1.2/ 0.8~1.2	-	~15000 spikes	Essential	~1.5	-	Effect of barrier layer
Z. Wang et al. *Nanoscale* 8, 14015 (2016).	SiO_2/TaO_x	Crossbar array	-	0.85~1/ 0.5~1	-	~6000 spikes	Essential	2~3	-	Single array
C. Liu et al. *J. Mater. Chem. C* 8, 12478 (2020).	HfO_2/HfO_x	Single cell junction	~6.25 %	0.8/ 1.6	-	-	Essential	2~2.5	-	Effect of V_o density
S. Kim et al. *ACS Appl. Mater. Interfaces* 9, 40420 (2017).	SiN_x/AlO_y	Single cell junction	-	5~6.15/ 4	-	-	Essential	~20	Achieved	CVD fabrication
A. S. Sokolov et al. *J. Alloys Compd.* 822, 153625 (2020).	TaO_x/IGZO	Single cell junction	~2.1 %	1.2/ 1~1.5	-	~250 spikes	Essential	~5	-	Surface modification
J.-H. Ryu et al. *Chaos Solit. Fractals* 140, 110236 (2020).	TiO_2/HfO_2	Single cell junction	-	8~10/ 5~9	-	~500 spikes	Not needed	~40	Achieved	Effect of barrier layer
L. Liu et al. *Adv. Electron. Mater.* 6, 1901012 (2020).	$HfO_2/BiFeO_3$	Crossbar array	-	0.72/ 1~1.5	100 % (9/9)	~500 spikes	Essential	2~2.5	-	3×3 array
J.-H. Ryu el al. *J. Alloys Compd.* 850, 156675 (2021).	Ta_2O_5/HfO_2	Single cell junction	~6.76 %	0.8~1/ 0.9~1.2	-	~1500 spikes	Essential	~12	-	Effect of barrier layer
C. Liu et al. *J. Phys. D: Appl. Phys.* 53, 035302 (2020).	AlO_x/HfO_x	Single cell junction	~7.14 %	0.5/ 1.4	-	-	Essential	~1.4	-	Switching mechanism
S. Liu et al. *Phys. Lett. A* 383, 125877 (2019).	AlO_x/TaO_x	Crossbar array	-	0.7/ 0.6~0.7	-	~2000 spikes	Essential	~5.3	-	10 device evaluation

Supporting Note 1. The effect of oxygen content variation on the TiO_x layer

The resistive switching characteristics of the TiO_x memristor devices originate from the formation/annihilation of electric-field driven oxygen vacancy (V_o) filaments in the TiO_x layer controlled by bias polarity, representing the redox-based switching property.[1-4] Because resistive switching is dependent on the controllability of the V_o filament, optimization of the V_o concentration in the oxide layer is required. Excessive V_o creation in low oxygen content below the lattice level could cause defect aggregation of the filament under repeated programming and could break the switching behavior itself, aggravating the switching reliability. Therefore, recent approaches to materializing TiO_x devices have mainly attempted to increase the oxygen content to obtain a stable switching operation by suppressing V_o aggregation.[5-7] However, at the same time, excessively lowering the V_o concentration could degrade the switching performance itself, such as the ON/OFF ratio and analog switching characteristic. From this point of view, the optimization of oxygen concentration ratio (x) in a TiO_x memristor that can alleviate the above trade-off issue is intensively required because the x value is related to the quantitative property of the V_o concentration in the memristive layer which plays a role as a basic unit in the conductive path controlled by the presynaptic voltage bias. Here, the TiO_x memristive layer formed by a direct sputtering method inevitably produces a lower V_o concentration at a high oxygen concentration ratio (x) of ~2.03 (Figure 1c), preventing the possibility of defect aggregation.

To investigate the effect of subsequent oxygen treatments on TiO_x stoichiometry, we executed a UV ozone (UVO) treatment after the TiO_x sputtering process and used reactive sputtering with a gas flow (Ar:O_2 = 20:4 sccm) to change the TiO_x deposition method. The changed material properties of Ti 2p and O 1s can be observed by X-ray photoelectron spectroscopy (XPS) (Figures. S1a and S1b). The spectra of the pristine TiO_x layer (x = ~2.03, black line) showed two main peaks (~457.9 eV and ~463.6 eV) in the Ti 2p spectra and a lattice oxygen peak (~529.3 eV) in the O 1s spectra, as in other papers.[1,8] After the UVO treatment (red line) and reactive sputtering (blue line), both Ti 2p and O 1s spectra slightly moved to the left (nonstoichiometric part) compared with the pristine TiO_x layer. Especially for the memristor device fabricated by the reactive sputtering method, the peak shift was more remarkable, and the relevant device entirely lost its resistive switching properties, as shown in Figure S1c (magenta line). Here, switching degradation was also observed in the UVO-treated TiO_x memristor device, and its degradation deteriorated when the duration of UVO treatment was increased from 1 to 10 min. This result indicates that excessive oxygen treatment beyond

the lattice value diminishes the switching performance due to a considerably deficient V_o concentration. Figure S1d shows the XPS depth-profiling analysis of the memristor device after UVO treatment for 10 min, presenting an x value of ~2.17 at the TiO_x switching layer, which is obviously distinguishable to the optimized value of ~2.03 (Figure 1c). As a result, we verified the theoretical basis that x value should approach to the lattice level of 2 for reliable resistive switching in the manner of array level fabrication and electrical investigation of the TiO_x memristor device.[1,5,7] This stoichiometric stabilization of the TiO_x memristive layer to prevent an excessive or a deficient V_o concentration provides the basis of large-scale passive memristor array to be constituent device element for hardware neural network system. In addition to this, V_o-based switching mechanism was also investigated by using transmission electron microscopic (TEM) analysis for each resistive state of the device as shown in Figure S1e. Here, the specific element distribution can be observed with intensity variation along with the vertical position for the OFF (left plot) and ON (middle plot) state. Compared to the OFF state, the oxygen intensity at ON state shows the broad distribution for the entire bulk region and shifts to the bottom direction, resulted from the negative voltage at the top Al electrode. In other word, the oxygen ions remarkably migrate to the active bulk region from top interface, which means lowering the top interface resistance. This feature is distinctly observed in comparison of oxygen intensity (right plot) for state transition from OFF (dashed line) to ON (solid line) state.

Supporting Note 2. The statistical estimation of device operational yield

Statistical results for switching threshold uniformity are presented for the device with 50 × 50 μm^2 of junction area (Figure S2a) and as an array-to-array variation (Figures S2b and S2c), indicating remarkable reliability of the TiO_x memristor array device to be used for constituent element in neuromorphic system. Based on the number of evaluating device cells, it is possible to extract operation yield for individual device cells. If all n devices are working on evaluation without failure at a fixed yield of x ($0 \leq x \leq 1$), the probability can be found as x^n, which denotes the probability density function of $y = x^n$ (Figure S2e). With this, the probability (P) for a specific range of x can be calculated, which is dependent on the n value. Then, the probability of yield beyond specific value of p can be calculated by conventional Bayes' theorem[9,10] as follows:

$$P(p \le x \le 1) = \int_p^1 x^n dx \Big/ \int_0^1 x^n dx = 1 - p^{n+1} \quad (1)$$

From $n = 2$ (black line) to $n = 120$ (green line), the curvature of x^n gets dramatically increased, resulting in increase of probability for the higher yield near ~100 % (colored box). When p is fixed, the P ($p \le x \le 1$) can be plotted as a function of n (Figure S2f), where the reliability for the device operational yield can be enhanced by the increased evaluation numbers. For example, the probability for yield ≥ 90 % reaches as ~100 % when n ≈ 60 (black line). Similarly, the probability values for yield ≥ 95, 98, and 99 % are close to ~100 % when $n \approx$ 120, 240, and 360, respectively. This indicates the statistical reliability of the TiO_x memristive device in the array level when sufficient number of device was evaluated showing switching property. In other words, the evaluated number of devices affects the reliability of device operational yield, and in our measurement procedure, more than 99 % of device yield can have assurance with high probability.

Supporting Note 3. Analog switching properties of the TiO_x memristor device

Figure S3a shows the PSC response of the TiO_x memristor device when the input pulse (V_p = −1.55 V for t_w = 150 ms) was sequentially accumulated over a period of Δt = 800 ms on the presynaptic neuron (top Al electrode). When we measured the PSC of the device at −0.5 V before and after a single V_p pulse train, the PSC gradually increased, indicating that the conductance through the V_o-based switching filament can be gradually increased by the potentiation pulse train. Additionally, the intermediate conductance states were well maintained before applying the subsequent V_p pulse (Figure S3a and Figure 1i), resulting in gradual uplift of the LTP process. Therefore, the PSC modulation behavior in this study successfully emulated an important function of biological synaptic plasticity.[11-13] Figure S3b shows the LTP/LTD characteristics of the TiO_x memristor device according to subsequent oxygen treatments (pristine, UVO of 1 and 10 min), where each 25 steps of potentiation pulse (V_p = −1.55 V) and depression pulse (V_d = 1.4 V) for t_w = 150 ms at Δt = 800 ms were applied. As discussed in Supporting Note 1, a reduction in the dynamic range was similarly observed after excessive UVO treatment on the TiO_x layer.

Supporting Note 4. Nonlinear pulse response of the TiO_x memristor device

As shown in the V_p variation for the LTP/LTD curves (Figure S5-1a and Figure 3e), a small change in pulse voltage can dramatically change the PSC value, that is, the nonlinear pulse response of the TiO_x memristor device. This nonlinear current transport, which depends on the magnitude of the V_p pulse, is minutely presented in the inset of Figure 1g, where ΔG_{avg} in the potentiation process nonlinearly decreased as V_p decreased. Indeed, the nonlinear pulse response is known to be a general feature in a redox-based TiO_x memory driven by V_o filament transport.[14] As the V_o filament is formed through the TiO_x layer in the potentiation pulse, the charge carrier can migrate along the neighboring V_o trap rather than via metallic transport.[3,5] Therefore, the major transport mechanism could be associated with the trap-assisted space-charge-limited conduction in the TiO_x layer, presenting a rapid reduction in the PSC at low potentiation voltage, expressed as $I \sim V^n$ (n ≈ 2), rather than ohmic behavior in conventional ECM transport.[8,15] According to this, the current value undergoes nonlinear voltage dependency, which in turn results in a significant reduction in the PSC at low potentiation voltage. Along with the reduction of unselected line transport in passive matrix array structure, the nonlinear characteristics are important for the remarkable modulation of the conductance change using a small variation in the potentiation voltage, which can be utilized to adjust the learning rate in a hardware-integrated neural network system (Figures 3e and 3f). Note that *I*-*V* nonlinearity does not affect the read process, because the binary input, which is represented either by 0 or V_R, is used for this work (see Methods).

Supporting Note 5. Detailed information on the hardware implementation and system automation

An analog switching matrix of a PCB board is implemented by MAX14661 16:2 and MAX14763 2:1 multiplexers. During the write and read programming,[16] the former selects the column and row of the target cell, while the latter selects the reference voltages to be connected (V_{RP}, V_{RN}, V_{SET}, V_{RST}, $V_{SET,H}$, or $V_{RST,H}$). Both MUXs are controlled by digital signals received from the FPGA board through a serial peripheral interface (SPI) bus. A read-out circuit is implemented by AD8542 as amplifiers of TIAs and ADS7952 as a 12-bit ADC. The resolution of the ADC is determined by (2).

$$R_{ADC} = R_{DAC} + R_{mem} + \log_2(Rows) \quad (2)$$

Here, R_{ADC}, R_{DAC}, R_{mem} are resolution of ADC, DAC and the conductance memristor, respectively. Since the theoretical value of R_{ADC} is 11.22, in this work, where R_{DAC}, R_{mem} and the number of rows are ternary, 5, and 25, respectively, we choose the resolution of the read-out ADC as 12-bit, which is sufficient for both training and inference. The control signal of ADS7952 and its output signal are transmitted by the SPI bus to the FPGA board. The input range of the ADC is within [0 V, 5 V], and the output code is produced in the [0, 4095] range. The reference voltages are produced by ADM7170 and ADM7151 regulators. The whole system is automated by a DE2i-150 FPGA board and PC. The test loop used to make the control signal for the MUXs and ADCs is executed by a MATLAB script. The FPGA board and PC communicate through UART serial communication with RS-232. The FPGA board consists of the UART interface, first-in first-out (FIFO) memory, an internal clock generator, and the SPI interface to the PCB board in VHDL. When the test loop starts, 584 bits (192 bits for 1 PCB board and 8 option bits) are transmitted to the FPGA, and serial data are stored in the FIFO memory and converted to the control signal of the MUX and ADC via digital signal processing units. After ADC conversion, the FPGA board receives a total of 480 bits (160 bits for 1 PCB board) of ADC output data from the PCB boards and transmits them to the PC via FIFO memory and UART communication.

Supporting Note 6. Clothes data set

To evaluate the training performance of the TiO_x memristor array device integrated in the hardware system, we tested the Clothes data set[17] via the same quantization process as used for the MNIST data set. As shown in Figures S12a and S12b, the analog magnitude (256-level) in each 28 × 28 pixel image was scaled down to 8-level resolution, and then the images were quantized as binary images (2-level) based on the median value in the digital computing platform. The output classes of the Clothes data set include shirt, trouser, pullover, dress, and coat, and the entire data set consists of 30,000 training images and 5,000 test images that are different from each other. The classification accuracy for the Clothes data set is presented in Figure S12c, where all the training processes are established by the standard backpropagation algorithm (see Methods). The result for the CNN hardware using the FCIS training (~82.3 %, red line) showed much better recognition performance than that for the single-layer simulation (~73.0 %, black line), indicating the availability of the TiO_x memristor device to the hardware neural network system. Figure S12d shows the reshaped 28 × 28 contour images

for the synaptic weight during the training process in the single-layer simulation, where the red pixels show enhanced weight (positively updated) and blue pixels show decreased weight (negatively updated), reflecting the common feature of sequential training as a relevant weight distribution.

Supporting Note 7. Quantitative analysis of in-situ training

The energy efficiency during inference is estimated in both hybrid analogue-digital peripheral circuits and memristor arrays. In a convolution module for a first layer, 50 × 5 size of kernel arrays performs 250 additions and the multiplications per an input pulse. During inference, since the time to take from input MUX control and to output A/D conversion is 28 μs as shown in Figure S13-1 and the 12-bit read-out A/D conversion, the throughput of convolution modules is T_{mem} is 50 × 5 × 4096 / 28 μs = 3.66 × 10^{10} operations per second (OPS) for 64 levels or 6-bit conductance of the memristor device. With 14.73 mW of the power consumption of a convolution module including the input MUX and read-out circuit (TIAs and ADC), the energy efficiency of a first layer convolution module is 2.48 TOPS/W. In addition, since the convolution modules for second layer has 50 × 20 sized memristor array and the three identical convolution modules are operated in pipelined manner, the energy efficiency of whole system is 4.13 TOPS/W. This performance figure shows nearly 41 times better energy efficiency when comparing to the metrics of Tesla V100 GPU (100 GOPS/W for 16-bit floating-point number computing). During training, it is hard to directly compare the energy efficiency of in-situ training on convolution module and the ex-situ training with digital processor, because there exist many different factors, such as the required number of iterations to reach the saturation point and the amount of reduced error per an iteration. Instead, the total energy consumption can be estimated through the simulation by modeling the quantitative indicator as power consumption per single device. Firstly, to model the training energy consumption on in-situ training of the convolution module and digital ex-situ training, the energy on energy consumption on multiplication and memory access should be quantified in in-situ training and ex-situ training, respectively. It is assumed that ex-situ training is conducted with 8-bit digital processor with 45 nm CMOS technology,[18] which consumes 0.2 pJ and 0.03 pJ of energy per single multiplication and addition, respectively. In the aspect of energy consumption on the weight update, since the memory access energy of DRAM is 20 pJ/bit, the total 160 pJ is consumed for the 8-bit weight update. On the other hand, in memristor case, when assuming the average of programmed conductance of a device is 1 μS, the energy consumption on multiplication calculated with a read pulse with 0.1 V of

read voltage and 1 μs of pulse duration is 10 fJ. When programming the conductance, the energy consumption on programming per a pulse with 2 V of voltage and 1 ms of pulse duration is 4 nJ. With the numerical figures of the training process in Figure S21a, training energy for same training epoch can be evaluated through the simulation as shown in Figures S21b and S21c. In order to compare only the energy consumed by operations of the memristor array with in-situ training, the energy consumed by interface circuits and other hardware area is ignored in the simulation. The results show that in-situ training with memristor array can significantly reduce the energy consumption on VMM computation by 11.7 times less than 8-bit digital processor over 10 epochs. In the aspect of programming energy, the energy consumption of programming with 10 ms pulse duration is larger than energy consumption on memory access of digital processor nearly 3.5 times, while that with 1 ms pulse duration is nearly comparable to the energy consumption of the 8-bit digital processor. In contrast, the FCIS training with 1 ms pulse duration consumes 2.8 times less energy on training than normal training with the same pulse duration due to the reduced number of programming pulses as shown in Figure 5c and Figure S16. This result implies that the technique to adjust amplitude of the programming pulse can efficiently increase the learning rate consuming relatively less energy than adjusting any other pulse conditions due to the nonlinear *I-V* characteristics of the device. To further improve the energy consumption on the training, we plan to improve device characteristics with respect to the pulse duration of the programming pulse, with keeping the uniformity characteristics for in-situ training in the future work.

In addition to the energy efficiency of the in-situ training, the tolerance for the device non-ideality of the in-situ training can be also quantified through the simulation. Figure S22 shows the performance improvement of in-situ training for device non-idealities; conductance variation with programming error, stuck-ON, and stuck-OFF. The simulation for programming error is assumed that the programming failure occurs as much as a percentage of the error in the target conductance change in the learning phase. The result in Figure S22a shows that in-situ training is more tolerant to the programming error than the transfer learning, in that despite a noticeable decrease in the classification accuracy of transfer learning when a programming error occurs up to 20 percent of target conductance change, in-situ training maintains the 94 % classification accuracy. The stuck-ON and stuck-OFF issues are device failure types that the conductance does not change under any programming conditions. These problems also introduce serious training error, especially in the transfer learning and several pre-ex-situ training techniques are have been proposed that include the device information on

ex-situ training before transferring. In the simulations, the stuck-ON and stuck-OFF devices are assumed to occur randomly in each column, which is represented in the *x*-axis of Figures S22b and S22c. The in-situ training maintains the classification accuracy over 95% until 6 of 25 devices under stuck-ON and stuck-OFF situation per column. These results show the advantages of in-situ training rather than the transfer learning not only for the realization of online learning but also the energy efficiency and device imperfection tolerance.

Supporting Note 8. The effect of conductance retention on in-situ training performance

The simulations are conducted under two different conditions; uniform retention and non-uniform retention, which represents whether the conductance ratio between analog states are constant depending the programmed conductance state or not. As shown in Figure 1i and Figures S23a and S23b, the retention characteristics of the memristor device is different depending on the programmed conductance state. To clarify the retention characteristics, the PSC ratio is introduced, which is a ratio of the conductance after retention divided by the initial conductance. Figure S23c shows the simulation result that all devices of CNN have uniform retention properties regardless of the conductance value. Since uniform conductance retention only affects overall conductance range while the ratio of conductance is maintained over the devices, the classification accuracy is recorded over 95 % despite of 60 % PSC ratio. In fact, because the VMM between input image data (read voltage) and encoded synaptic weight (memristor conductance) has importance for their 'relative' proportion and ordering of output activation function values, the absolute magnification is not the critical factor for the artificial neural network system. In this simulation, the PSC ratio in Figure S23a is applied as an average value of all conductance states, which is ~56.2 %, and 95.5 % classification accuracy is recorded. In non-uniform retention simulation, the PSC ratio is applied to the conductance differently according to the conductance range as a normalized ratio, and the degree of the non-uniformity is evaluated by the minimum value of the normalized ratio. The simulation result of measured retention characteristics of the device is close to the result that the minimum value of normalized ratio is 0.8 at 95.2 % of classification accuracy. In addition, when the PSC ratio is reduced as the retention becomes worse after a few days, the degraded conductance states can be retrained. As shown in Figure S23e, we actually performed the hardware neuromorphic training process with 'long time interval' after the suspension of training step. During the training process, when the iteration number reached 3,000, we

stopped all training process and left the situation as it is while 3 days (~72 hours). After that, we restarted it and observed that loss function was well decreased regardless of 3 days of memristor device retention time. The loss function value at 4,000th iteration was measured as 0.07, which is corresponded to the accuracy of ~95.2 % based on results in Figures 5a and b (main text). Therefore, we concluded that the synaptic weight map of memristor conductance configuration is well maintained even in 3 days of retention time enough to present the original accuracy, indicating tolerance for retention property of in-situ training.

Supporting Note 9. Toward further sophisticated optimizer: RMSprop

The proposed FCIS training is a simple form of the learning rate optimization technique. The ultimate goal of learning rate optimization in device level is applying the sophisticated optimization technique such as AdaGrad or RMSprop. Since those adaptive optimizers adjust the learning rate for each weight separately according to the amount of update in weight in the last iteration, the optimizers can be solution to learning problem, such as local minima, which can occur due to the imperfection of the device. For example, in RMSprop, the learning rate is adjusted as follows:[19]

$$G_t = \gamma G_{t-1} + (1-\gamma)(\nabla_w J(w_t))^2 \tag{3}$$

$$w_{t+1} = w_t - \frac{\eta}{\sqrt{G_t + \varepsilon}} \cdot \nabla_w J(w_t) \tag{4}$$

where γ is called a decaying factor, of which value is usually set between 0.9 and 0.999 and ∇J is a gradient matrix of backpropagation. If it is assumed that the computation in (3) be achieved in learning processor in the digital domain, the adjusted learning rates of each device can be applied in in-situ training as the amplitudes of SET voltage as shown in Figure S24. This scheme enables to update the weight not only by the number of the pulses, but also by the amplitude of the pulse reflecting the adjusted learning rate for individual devices. One of the other advantages of the learning rate optimizer besides the fast convergence is the further tolerance to the device imperfection than normal in-situ training. The effect of the RMSprop optimizer for the device imperfections is verified by simulation as shown in Figure S25. Those results show that RMSprop can provide more fast convergence to the target performance or better training accuracy rather than normal training.

Supporting Note 10. Feasibility of backpropagation using convolution module

As shown in Figure S26-1a, the convolution module can be utilized for the backpropagation of the error function as well as forward propagation. The forward and back propagation results for each layer are represented by O and δ. From the equations (5)-(6) in the main text, the amount of weight update in 2nd layer is determined by the inner product of δ, derivative of sigmoid function O from the 2nd layer and O form the 1st layer. In order to produce the δ, the function of backward propagation is implemented by using the analogue MUXs as backpropagated inputs, which are connected to the column of the memristor array, and connecting the neuron circuits to the rows of the memristor via column selectors. The results of backpropagation through memristor array is shown in Figure S27 with comparison to the simulation results for the same input pattern. It shows the feasibility of the backpropagation in hardware-level and the possibility of the fully hardware implementation of backpropagation learning if the learning processor can be efficiently implemented within hardware.

Supporting Video.

Description: The in-situ training process on the MNIST image data set for the memristive hardware system. The left and right panels represent the FCIS training and normal in-situ training, respectively. Among the randomly selected input batches, a representative image is shown at the upper-left position in each panel along the training iteration. The image at the upper-right position in the panel shows the 1st feature map for example image “2”. The feature map changes from dim to distinct image as the training progresses from the initial iteration. The image “2” is inferred after every weight updating of one iteration is completed. Finally, the image at the lower position shows the change in the loss function with the training iteration.

References

[1] K. Szot, M. Rogala, W. Speier, Z. Klusek, A. Besmehn, R. Waser, *Nanotechnology* **2011**, *22*, 254001.

[2] S. G. Park, B. Magyari-Kope, Y. Nishi, *IEEE Electron Device Lett.* **2011**, *32*, 197.

[3] H. Y. Jeong, S. K. Kim, J. Y. Lee, S. Y. Choi, *Appl. Phys. A* **2011**, *102*, 967.

[4] H. Y. Jeong, J. Y. Lee, S. Y. Choi, J. W. Kim, *Appl. Phys. Lett.* **2009**, *95*, 162108.

[5] S.-J. Park, J.-P. Lee, J. S. Jang, H. Rhu, H. Yu, B. Y. You, C. S. Kim, K. J. Kim, Y. J. Cho, S. Baik, W. Lee, *Nanotechnology* **2013**, *24*, 295202.

[6] W.-Y. Chang, Y.-T. Ho, T.-C. Hsu, F. Chen, M.-J. Tsai, T.-B. Wu, *Electrochem. Solid State Lett.* **2009**, *12*, H135.

[7] P. Bousoulas, I. Michelakaki, D. Tsoukalas, *J. Appl. Phys.* **2014**, *115*, 034516.

[8] J. Zhao, M. Zhang, S. Wan, Z. Yang, C. S. Hwang, *ACS Appl. Mater. Interfaces* **2018**, *10*, 1828.

[9] B. Efron, *Science* **2013**, *340*, 1177.

[10] D. L. Faigman, A. J. Baglioni, *Law Hum. Behav.* **1988**, *12*, 1.

[11] S. H. Jo, T. Chang, I. Ebong, B. B. Bhadviya, P. Mazumder, W. Lu, *Nano Lett.* **2010**, *10*, 1297.

[12] J. Jiang, J. Guo, X. Wan, Y. Yang, H. Xie, D. Niu, J. Yang, J. He, Y. Gao, Q. Wan, *Small* **2017**, *13*, 1700933.

[13] M. Zhao, B. Gao, J. Tang, H. Qian, H. Wu, *Appl. Phys. Rev.* **2020**, *7*, 011301.

[14] E. Lim, R. Ismail, *Electronics* **2015**, *4*, 586.

[15] R. Waser, R. Dittmann, G. Staikov, K. Szot, *Adv. Mater.* **2009**, *21*, 2632.

[16] P. Yao, H. Wu, B. Gao, S. B. Eryilmaz, X. Huang, W. Zhang, Q. Zhang, N. Deng, L. Shi, H.-S. P. Wong, H. Qian, *Nat. Commun.* **2017**, *8*, 15199.

[17] H. Xiao, K. Rasul, R. Vollgraf, **2017**, *Preprint at https://arxiv.org/abs/1708.07747*.

[18] M. Horowitz, **2014**, *IEEE Int. Solid-State Circuits Conf. Digest of Technical Papers (ISSCC), IEEE*, Piscataway, NJ, pp. 10.

[19] T. Tieleman, G. Hinton, **2012**, “Lecture 6.5—RmsProp: Divide the gradient by a running average of its recent magnitude” COURSERA: Neural Networks for Machine Learning.
